# Synthesis Attempt and Structural Studies of Novel A_2_CeWO_6_ Double Perovskites (A^2+^ = Ba, Ca) in and outside of Ambient Conditions

**DOI:** 10.1021/acsomega.2c00669

**Published:** 2022-05-23

**Authors:** Damian Wlodarczyk, Mikolaj Amilusik, Katarzyna M. Kosyl, Maciej Chrunik, Krystyna Lawniczak-Jablonska, Michal Strankowski, Marcin Zajac, Volodymyr Tsiumra, Aneta Grochot, Anna Reszka, Andrzej Suchocki, Tomasz Giela, Przemyslaw Iwanowski, Michal Bockowski, Hanka Przybylinska

**Affiliations:** † Institute of Physics, Polish Academy of Sciences, Ave. Lotnikow 32/46, PL-02668 Warsaw, Poland; ‡ Institute of High Pressure, Polish Academy of Sciences, Sokolowska 29/37, PL-01142 Warsaw, Poland; § Military University of Technology, Gen. Sylwestra Kaliskiego 2, PL-00908 Warsaw, Poland; ∥ Chemical Faculty, Gdansk University of Technology, G. Narutowicza 11/12, PL-80233 Gdansk, Poland; ⊥ Solaris Synchrotron NSRC, Jagiellonian University, Czerwone Maki 98, PL-30392 Cracow, Poland

## Abstract

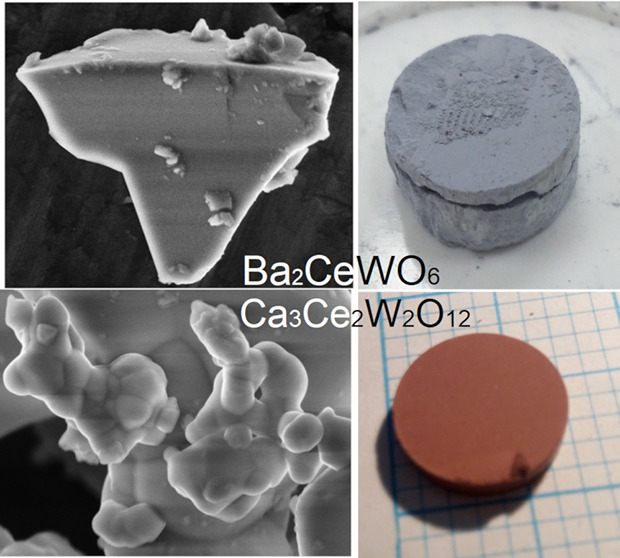

This comprehensive work showcases two novel, rock-salt-type minerals in the form of amphoteric cerium–tungstate double perovskite and ilmenite powders created via a high-temperature solid-state reaction in inert gases. The presented studies have fundamental meaning and will mainly focus on a detailed synthesis description of undoped structures, researching their possible polymorphism in various conditions and hinting at some nontrivial physicochemical properties like charge transfer for upcoming optical studies after eventual doping with selectively chosen rare-earth ions. The formerly mentioned, targeted A_2_BB′X_6_ group of compounds contains mainly divalent alkali cations in the form of ^XII^A = Ba^2+^, Ca^2+^ sharing, here, oxygen-arranged clusters (^II^X = O^2–^) with purposely selected central ions from f-block ^VI^B = Ce^4/3+^ and d-block ^VI^B′ = W^4/5/6+^ since together they often possess some exotic properties that could be tuned and implemented into futuristic equipment like sensors or energy converters. Techniques like powder XRD, XPS, XAS, EPR, Raman, and FTIR spectroscopies alongside DSC and TG were involved with an intent to thoroughly describe any possible changes within these materials. Mainly, to have a full prospect of any desirable or undesirable phenomena before diving into more complicated subjects like: energy or charge transfer in low temperatures; to reveal whether or not the huge angular tilting generates large enough dislocations within the material’s unit cell to change its initial properties; or if temperature and pressure stimuli are responsible for any phase transitions and eventual, irreversible decomposition.

## 1 Introduction

Double perovskites (DPs) are known as rock-salt type minerals named after the famous Russian mineralogist Lev Perovski.^1^ They resemble a typical ABX_3_ perovskite structure but with one simple exception: they have double-sized unit cells having a general A_2_BB′X_6_ formula. They are ordered in such a fashion that one block shares a corner arrangement with both unique BX_6_ and B′X_6_ clusters.^2^ A-site cations are mostly chosen as mono-, di-, or even trivalent alkaline metals—in our case, Ba^2+^ and Ca^2+^ ions. B and B′ sites are suitable combinations of rare-earth or transition type atoms, such as cerium (Ce) and tungsten (W). Having an overview of all known perovskites, one must note that X could stand for halides, nitrides, and sulfides shaped artificially after the exact properties that are truly desired. In our case, they would be in a form of more environmentally friendly oxides (O^2–^) since we aim to check whether or not these newly coupled tungsten–cerate matrices will be good enough to host a stable, luminescence-active environment. They may be suitable for near-ultraviolet – near-infrared (NUV–NIR) down-conversion knowing that most of these compounds burn and decompose while being illuminated.^3−5^ In addition, they could be used for the construction of widely known UV–X-ray sensors with reversible charge-transfer capabilities before doping with some other rare-earth ions such as Pr, Eu, or Yb^3+^.^6,7^ These dopants could facilitate some properties toward thermometry in cryogenic temperatures.^8^ We based our hopes on previously studied scintillating material, BaWO_4_ doped with cerium,^9^ which served as a fundamental background, an inspiration due to its composition’s similarity and interesting polymorphism. This popular scintillator had the potential for reinforced energy-transfer phenomena which could be useful in creating renown down converters. We have also calculated promising Goldschmidt factors which are popular, theoretical modeling parameters for DPs (with ∼74% accuracy).^10^ Here, they are placed conveniently in the tolerance region between 0.825 and 1.059, ideally close to 1 for barium compounds between ∼0.989 and 0.967 and calcium compounds between 0.899 and 0.879 depending on which BB′ central ion pair is chosen from the mix — Ce^4+^/W^4+^ or rarer Ce^3+^/W^5+^. One can assume successful synthesis and product placement of those materials in at least one of the aforementioned fields even after comparing those numbers to the newly modified law of Bartel et al.^11^ This modernized GS (mGS) factor does not change much in terms of estimates provided earlier, but on the basis of modern advances in technology and overall statistics collected after nearly a century, it is more accurate (∼92%) and rigorous (*r*_A_ > *r*_B_; mGS < 4.18, the closer to 0 the better) as can be seen in Table 1.

**Table 1:** Classic (GS)^10^ and Modernized (mGS)^11^ Goldschmidt Parameters Fashioned into Calculating Theoretical Factors Predicting the Existence of Discussed, Newly Developed Materials

**Ionic radii (Å)**				**Goldschmidt Factors**	
**^XII^A^2+^**	**^VI^B-site (Ce)**	**^VI^B′-site (W)**	**^II^X (O^2–^)**	***t* (GS)**	**τ (mGS)**
Ba 1.61	Ce^4+^ 0.87	W^4+^ 0.66	1.35	0.989	3.421
	Ce^3+^ 1.01	W^5+^ 0.62		0.967	3.460
Ca 1.34	Ce^4+^ 0.87	W^5+^ 0.62		0.908	3.940
	Ce^3+^ 1.01	W^6+^ 0.60		0.883	4.210

Of course, many other abilities could emerge during this research. Since early 1950, this general group of materials was constantly investigated and systematically subcategorized due to their broad chemical flexibility. After the development of new analytical techniques, we now know that several types of atoms could be incorporated into these compounds and simply change their optoelectronic and magnetic properties.^3^ Moreover, they can host different electrical properties ranging from insulating, half-metallic, to even metallic.^12,13^ Some might even show spin-polarized electrical conductivity or superconductivity.^14−17^ Quite a few develop magnetic ordering^18,19^ ranging from para-, antiferro-,^20,21^ and even ferrimagnetic,^22,23^ sometimes accompanied by spontaneous geomagnetic resistivity,^13,24^ frustration,^25,26^ or eventual superexchange interactions.^27−29^ This occurs especially within the matrices hosting long-range A^2+^-3d^n^-O^2–^-2p^6^-W^6+^-5d^0^ orbital couplings.^19,30^ Adjustable conductivity^31^ and eventual photocatalytic properties are also within grasping range.^32−34^ In general, these materials have gained great technological interest as dielectrics^35−37^ or piezoelectric sensors,^8,38,39^ magnetic memory components,^13^ electrodes, and media for well-developed fuel-^40−42^ or solar cells.^43−45^ But this is a topic for a separate discussion and will not be divulged here further.

## 2 Experimental Section

### 2.1 Instrumentation Parameters

Powder X-ray diffraction (XRD) data were collected on an X’pert MPD (Panalytical) diffractometer equipped with a Cu (Kα) lamp (40 kV, 30 mA) involving a Johansson-type germanium monochromator and a semiconducting strip-detector of resolution close to 0.05°. Broad patterns (from 10 up to 150° 2θ) were collected within 3 to 12 h with a setup working in Bragg–Brentano geometry. Rietveld refinement was performed using the FullProf Suite.^46^

Confocal micro-Raman spectra were registered on MonovistaCRS+ spectrometer provided by S&I Gmbh having a 0.75 m Acton-Princeton monochromator; back-thinned, deep-depleted PyLoN system, nitrogen-cooled (−125 °C) CCD camera (1340 × 100–20 μm per pixel array); computer-controlled Olympus XYZ IX71 inverted stage equipped with Moticam (1280 × 1204 camera) and long working distance objectives such as 50× (NA 0.9, ambient conditions), 10× (NA 0.23, high-pressures) and 5× (NA 0.12, low temperatures) magnifications used during various studies. Lasers used after notch-filtering (∼60 rel. cm^–1^) and gray-filter power manipulation were (automated by Trivista software): green, diode-pumped Cobolt Samba 04-01 series emitting 532 nm wavelength through 2400 grooves per mm holographic grating; and NIR Torsana StarBright L series 785 nm laser alongside with 1500 grooves per mm grating. The entrance slit had 100 μm and the final resolution was 0.88 cm^–1^ for green, 0.49 cm^–1^ for NIR laser, respectively. The acquisition time was substantially long (from 40 min to 2h) since both powdery samples strongly absorb light and can overheat quickly — in order not to burn both samples power density was set within the range of 0.217÷1.12 mW/μm^2^ depending on the user setup and applied conditions.

Complementary Fourier transform infrared (FTIR) spectra were collected using a Nicolet 8700 spectrometer from ThermoElectron Corp. working in Attenuated Total Reflectance mode. 64 scans, 1 s each was gained at a wide range of 400–4500 cm^–1^ with resolution set around 1 cm^–1^.

Electron paramagnetic resonance (EPR) data were acquired via Bruker ESR300 spectrometer working in X band (9.5 GHz), with precision around 100 mG in between 1 to 8.6 kGauss. Powders were placed in 4 mm thin wall quartz tubes of 250 mm length provided by WilmadLabGlass through Merck SA.

Synchrotron radiation experiments—X-ray Absorption Spectroscopy (XAS)—were performed on pellets at the SOLARIS NSRC facility in Cracow, Poland. The bending magnet (1.31 T) at the BL04 beamline was used in the Total Electron Yield detection mode. The synchrotron worked at 1.50 GeV. Ultrahigh vacuum was applied ∼1.5 × 10^–9^ mbar. The beam spot size was roughly 2.5 mm in diameter. No specific polarization was used. The resolution was set in between 75 (Ca L_2,3_-edge, O K-edge) to 150 meV (Ba, Ce M_4,5_-edges) depending on the choice of specific edge. The total working range was 150–1700 eV. Samples in the form of compressed (2 tons) pellets were placed on Mo holders and tightened using screws and pins. Background measurements on empty holders were also performed as a reference.

X-ray photoelectron spectroscopy (XPS) measurements were performed after XAS in a Prevac setup equipped with a Scienta R4000 hemispherical analyzer (pass energy 200 eV) and monochromatic X-ray tube (Al Kα −1486.7 eV). The full width at half-maximum (fwhm) of the 4f_7/2_ Au line measured at the same experimental condition was 0.6 eV. The orbitals O 1s (K), and W 4f (N_6,7_), Ce 3d (M_4,5_), Ba 3d (M_4,5_), or Ca 2p (L_2,3_), spin–orbit doublets were measured. Spectra were analyzed using the commercial CASA XPS software package (Casa Software Ltd., version 2.3.17) with mostly Shirley not Tougaard background. Spectra were fitted with a mixed Gaussian–Lorentzian function. Mounting and proper electrical contact between low-conducting powder and the instrument was ensured by the incorporation of carbon tape.

Differential scanning calorimetry (DSC) and heat capacity (Cp) data were taken on a Netzsch Phoenix DSC apparatus, model DSC 204 F1. The temperature scan ranged from room conditions to 873 K at a heating rate of 10 K per min. A three-staged, heating→cooling→heating system was set in inert gas→inert gas→air conditions to assess the particular behavior of each sample in a specific environment. The Cp reference was saphire. Crucibles hosting investigated powders were made out of concave Al pans.

Thermogravimetry (TG) was carried out subsequently in nitrogen and air conditions (20 mL per min of nitrogen flow) using Netzsch Tarsus, model TG 209 F3, at temperatures ranging from 293 to 1273 K. The heating/cooling rate was roughly 10 K per min. The mass of each powdery sample placed in a small corundum crucible was approximately 10 mg.

A Hitachi SU-70 SFE scanning electron microscope equipped with dispersive radiation detector (EDX) and cathodoluminescence system GATAN Mono CL3 was used for taking SEM images. Powders were additionally processed for statistical purposes using ImageJ software.

High-pressure (HP) measurements were performed up to 20 GPa via hydraulic press in Almax easyLab diamond anvil cell (DAC) using 0.45 mm, II-as type culet. The applied pressure transmitting medium (PTM) was argon, and ruby was used as a pressure gauge. Gaskets with 0.15 mm holes were made from Inconel x750 alloy and filled up to 50% with powders.

Low temperature (LT ∼5 K) measurements were performed in continuous helium-flow, Oxford Instruments cryostat model CF1204. High temperature (up to 873 K) data were collected in an inert (N_2_) and air atmosphere using LINKAM FTIR600 stage working together with T95 controller and LNP95 water pump operated via PC LINK software. Both instruments were equipped with quartz windows.

### 2.2 Synthesis Methodology

Both materials (powders) were synthesized using high temperatures in an oxygen-depleted/airless atmosphere via a solid-state reaction in constant-flow reactors up to 1373 K. Initially, the pressure was lowered to 0.7 atm to improve the flow and desorption of gas coming out of the decomposing fine powder reactants pressed into pellets (*d* ∼ 13 mm) placed in corundum crucibles after grinding in ethanol at each and every step. Later, the chamber was pressurized to ∼200 atm to increase the ionic diffusion rate of certain ions and ensure that the oxygen was flushed out of the reactor.

**Figure 1:**
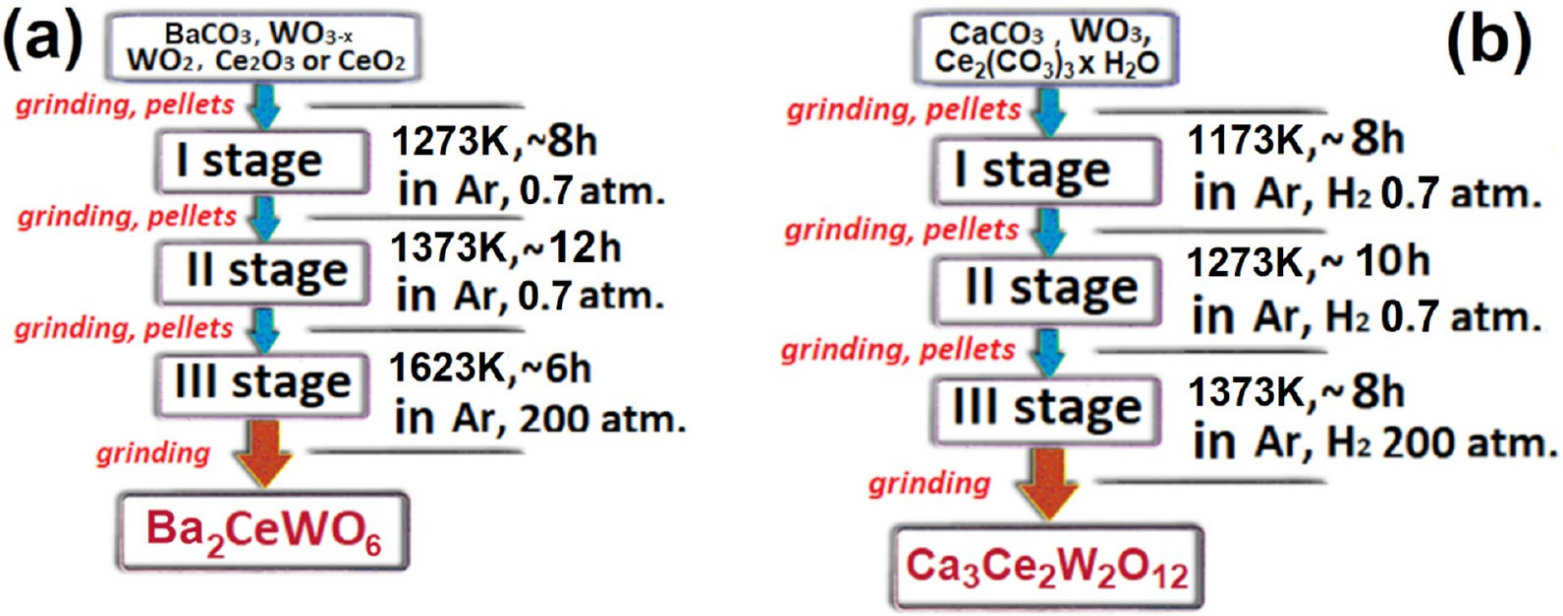
Overview schemes depicting detailed information about each synthesis stage for both (a) BCW and (b) CCWO compounds, respectively.

The final version of the Ba_2_CeWO_6_ batch (BCW–Ce^4+/^W^4+^ pair type) was made of 99.999% pure BaCO_3_, CeO_2_ 99.995%, and WO_2_ 99.9% pure (0.1% was metallic tungsten, identified by the user via XRD) purchased from STREM Chemicals, Inc. To protect W^4+^ from oxidation above 823 K, an inert atmosphere of argon (99.9996%) was incorporated (Linde, Corp.) after passing through a gas filtration system. Three-stage synthesis ensued on this incongruent sinter as depicted in Figure 1a and described below:
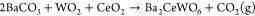
a
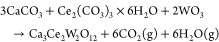
b

During the first stage (I) of BCW synthesis, one can also use other reactants like (NH_4_)_2_Ce(NO_3_)_6_ (from Merck) instead of CeO_2_ for a similar outcome but must be aware of two intermittent (∼15 min long) stops to be made at 723 and 1173 K due to a big release of H_2_O, NH_3_, NO_*x*_, and CO_2_, respectively. The heating rate of ∼10 K/min should not be exceeded; stages II and III should be followed by a rapid cool down via simple shutdown of the heater while maintaining constant Ar flow all the time—until at least 323 K to protect the reactants; stage III is something else—the material is sealed in a high pressure (200 atm) chamber without the constant flow of Ar gas. A ±10 K temperature balance is tolerated. Heat-shock in Ar is also valid. One should not overheat the pellet above 1663 K or otherwise, BaWO_4_ will form rapidly and excessive WO_2_ evaporation will commence peaking at 1723 K. A slight surplus of WO_2_ 1–2.5% mass or even more elevated gas pressure, might help mitigate that phenomena present above 1373 K. If not amended at all, it will lead to excessive BaO and CeO_2-x_ distorted oxides lingering on the surface.

As for the calcium sample (Ce^4+^/W^4+^ pair type), synthesized with the same CeO_2_ and WO_2_ ingredients and 99.95% pure CaCO_3_ powder from STREM Chemicals, it turned out to form a double-phase material: CaWO_4_ scheelite and Ca_3_Ce_2_W_2_O_12_ ilmenite-like compound due to case-specific mismatch by internal, structural, monoaxial tilting (mostly one angle in [001] direction) and some uncontrolled high-temperature charge-transfer between Ce and W atoms. Since CaWO_4_ is already a well-known material,^47−49^ we have decided to synthesize and describe the pure, novel Ca_3_Ce_2_W_2_O_12_ (CCWO) as a side-effect, rhombohedral phase because, according to Vasala and Karpinen,^3^ unreported ilmenite-like structures carry also some significance for scientists.^50^ They are very popular byproducts achieved after unsuccessful DPs synthesis attempts, especially if the prognosed GS factor is too low (<0.89). To see how our products statistically place themselves among others, already-synthesized compounds, see the modified Vasala and Karpinen^3^ charts in the Figure S1.

To meet the proper stoichiometry of the aforementioned compound in its purest form one must follow a crudely depicted process in Figure 1b and use CaCO_3_ 99.95% pure, ∼99% Ce_2_(CO_3_)_3_ × n (∼6) H_2_O, and 99.995% pure WO_3_ provided in our case also by STREM Chemicals, Inc. Intercalated water in cerium(III) carbonate was assessed via TG and powder XRD patterns to show that it has mostly ×6H_2_O with CeOH(CO_3_) and a little bit of (∼1%) Ce(CO_3_)_2_. Sintering stages included a slightly modified atmosphere in comparison to BCW: (I) Ar:H_2_ mixture in 999:1 mL ratio; (II) 997:3 mL; and (III) final Ar:H_2_ 995:5 mL flow. The heating rate was also about 10 K/min, and analogical temperature stops were applied during the first annealing. A fast cooldown via furnace shut down was still maintained but below 1273 K. The Ar:H atmosphere was kept till 323 K despite CCWO behaving more congruently. Slow cooling was proven to favor AWO_4_ and A_2_WO_5_ phase formation leaving highly distorted, inflated cubic CeO_2–*x*_ mostly in an unbonded state. Such high temperatures were still essential to ensure a proper Ce migration rate which is dictated by Tamman’s rule^51^ and cerium(IV) oxide’s high melting point (2400 °C). However, in the case of Ce^3+^, it is not as drastic (2177 °C). Extensive AWO_4_ evaporation occurs at the point above 1700 K so it is not advised to heat any higher since this product will uncontrollably sublimate from the pellet.

Because of problematic issues regarding paired, dual cationic structures one can also choose to synthesize those double perovskites or ilmenite-like structures using other materials while maintaining 9+ or 8+ BB′-site charge balance, respectively. Namely, CCWO preferring a 9+ balance could be synthesized containing in that particular case Ce^4+^/W^5+^, not Ce^3+^/W^6+^, and BCW could be made of a different Ce^3+^/W^5+^ 8+ pair cations, not Ce^4+^/W^4+^. Our team tried that approach to check whether it will be possible to omit the presence of both ions and just have one ion of each Ce/W instead. Unfortunately, it was not achieved; the ratio of BB′-site pairs changed even with Ar:H_2_ gas input and spontaneously occurring disproportionation was unavoidable.

Some attempts were made to synthesize those materials using Pechini, hydro-thermal/solvothermal methods since they are much cleaner and provide better control and homogeneity, but several issues led to a quick rejection of all undertaken attempts: First, during calcination and drying in air, materials oxidized beyond desired proportions, especially when polymer resin (mannitol) had to be thoroughly removed. Second, water is also a bad environment since it hosts a substantial amount of dissolved or in-build oxygen. Furthermore, in terms of protecting vital, not-fully oxidized, amphoteric reactants like Ce^3+^ carbonate, WO_2,_ or W_18_O_49_, an acid needs to be added at some point which often leads to even more pronounced oxidation. Third, using any good organic, alkalized (NaOH), alcohol mixture with surfactants (like EDTA or DTPA) under mild temperatures (considering being stored below 180–200°C in a pressurized autoclave to 6–8 atm) inevitably always resulted in the formation of high 20–30% content of other, highly undesired luminescent-active impurities like A_2_WO_4_, A_2_WO_5_, or even AWO_4_ mixed with (A = Ba/Ca^2+^) cerates or simple ceria rendering further experiments useless. That led the team to believe that actual, slow migration control of well-mixed components during pressurized solid-state reaction in an inert atmosphere is the key.

A previously mentioned component, hosting W^5+^ ions, could be made according to the recipe from the articles.^52−55^ W_18_O_49_ has the best 4+/5+ ratio of ions in the whole WO_3–*x*_ family. Also known as WO_2.72_, made by Fita-Chala et al.,^52^ it has the best properties according to our experience. It is the closest formula to a nonexistent, perfect 5+ W_2_O_5_/WO_2.5_ oxide. It is important to note that this compound is relatively unstable with time - slow oxidation outside of suspension and protective environment to W^6+^ is imminent. Thus, we recommend using it no longer than 3–4 days after synthesis. W_2_Cl_10_ is not a good solution either since it is stable but would contaminate structures with persistent chlorides. Because of those problematic issues, we refrained from studying the family of samples made out of these oxides and solely focused on those from Ce^4+^/W^4+^ substrates in terms of BCW, and Ce^3+^/W^6+^ regarding CCWO. As one can see later, either way, the presence of these pairs is still unavoidable.

## 3 Results and Discussion

### 3.1 SEM Imaging and Morphology

**Figure 2:**
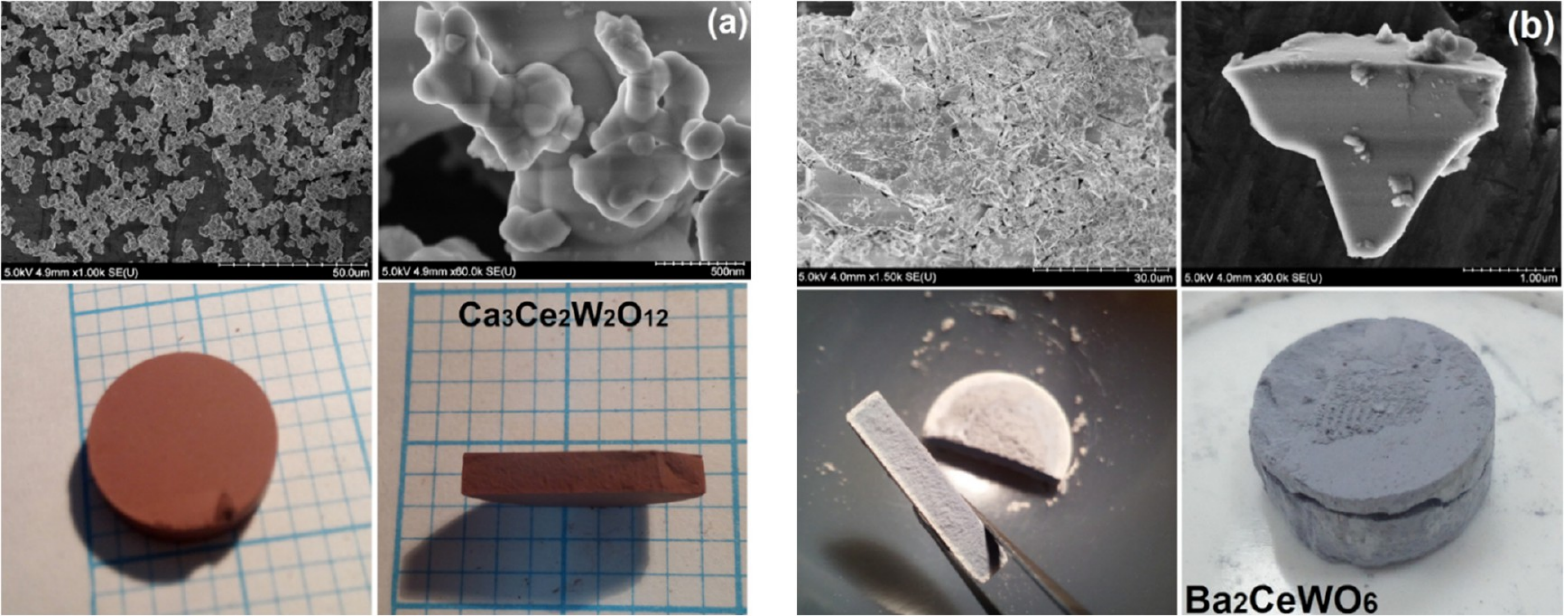
Micro (upper) and macroscopic (lower) photos of newly created materials: (a) homogeneously brown CCWO ilmenite, and (b) grayish-blue BCW double perovskite. Note that BCW has a more defined microcrystalline structure, and depending on the conditions, some crucial ingredients like WO_2_ can evaporate from its surface creating an unwanted white, brittle layer of (BaO+CeO_2-x_) on the outside.

The micro and macro visualization of both samples’ final forms post solid-state reaction is depicted in Figure 2 where one can see that CCWO (Figure 2a) has a chestnut-brown color and it is more in a form of widely spread micro-nano-sized spherical particles cluttered strongly together as fine dust. BCW, on the other hand, is a little bit smaller and has a steel-gray color crystallite polygons as shown in Figure 2b. Statistics done in ImageJ software on randomly chosen 20 grains show that the average size for CCWO particles is about 1035.81 ± 114.71 nm with widespread results ranging from 802 to 1220 nm, while for BCW the mean value is 781.31 ± 95.55 nm considering particles in between 461 and 1428 nm. The Debey-Scherrer equation could also provide some insight into the subject based on already completed XRD patterns.^56^ However, for grains larger than 200 nm the estimate is less accurate. So, based on shape constants (*K*) derived from three chosen, well-distinguished peaks, their *hkl’s*, and fwhm’s: in BCW (*K* ∼ 0.88–0.90;^57^ at 2Θ = 140.0, 92.5, and 69.9) the average size would be 861.61 ± 48.00 nm and for CCWO (*K* ∼ 0.84–0.86;^57^ and 2Θ = 77.2, 44.6 and 31.9) approximately 915.96 ± 17.77 nm.

BCW can occasionally form dark-gray crystals having a violet tinge inside the tightly pressed pellet. If overheated in the air—without protective, inert Ar—both materials will decompose to yellowish-white powders, respectively, as shown in Figure S2. Their composition and mechanisms of slow oxidation and WO_2_ evaporation will be discussed in much more detail later exploring DSC/TG and XRD sections. But, for now, one can be certain that in such high temperatures, BCW behaves as an incongruent mixture that can create brighter, unwanted outer layer shells on a pellet enriched in highly deformed, unintegrated, cubic CeO_2–*x*_ and/or sometimes even scattered BaWO_4_ grains. CCWO possesses a more congruent character and does not experience such prominent discoloring on the surface after extensive, inert heating. One certainly must look out for carbon contamination and avoid using graphite crucibles at higher temperatures since slowly evaporating W could bond with C creating a thin, darker film or pieces of W/WC ceramics. This dark brown hue is, however, easily removable with a simple touch from a piece of cloth, scalpel or tweezers under the microscope.

### 3.2 Powder XRD Patterns in Ambient Conditions

**Figure 3:**
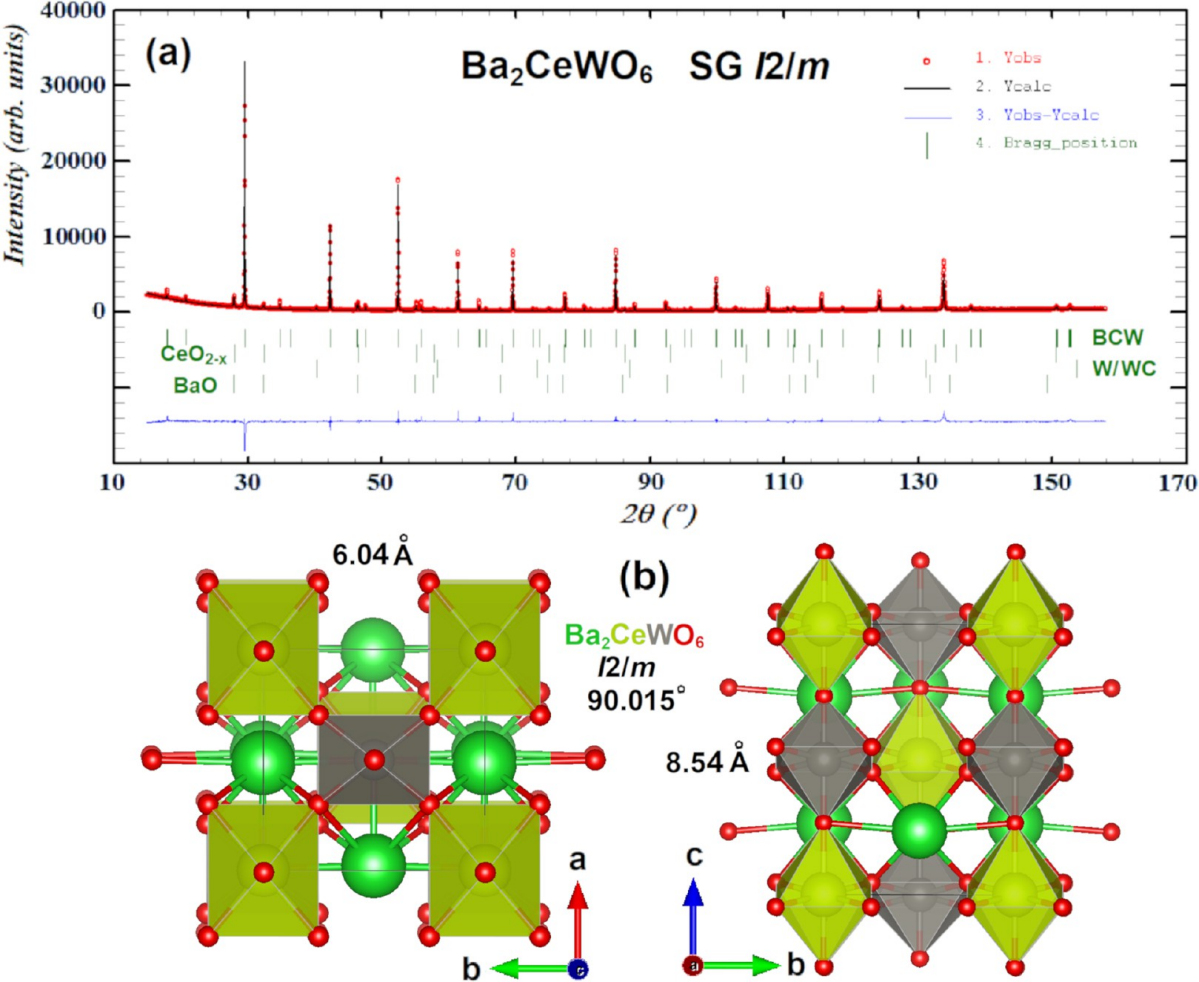
Original XRD pattern (a) after Rietveld fitting and refinement of best, 95% pure *I*2/*m* phase containing traces of BaO and highly distorted CeO_2-x_ (probably Ce_7_O_12_) oxides alongside with minor traces of W/WC phase. Red dots are the manifestation of the real, observed pattern, the straight black line is the calculated fit, and the blue line depicts the difference between them. Green, vertical lines are specific Bragg positions assigned to specific phases. Unit cell in partial ball–stick, partial polyhedral filling convention is shown in figure (b) along “c” (left) and “a” (right) axes. Yellow spheres and polygons are ceria, red is oxygen, and gray is tungsten. Barium is depicted solely as green spheres to improve overall visibility since its dodecahedra cover most of the view.

The pure Ba_2_CeWO_6_ XRD diffractogram, presented in Figure 3a, highly resembles a Ba_2_BiYO_6_ ICSD 65555 pattern from the work of Lenz et al.,^58^ exhibiting a *Fm*-3m space group (SG). However, Rietveld refinement has shown unsatisfying results regarding peak intensities and shapes. Ratios were clearly underestimated, which led to a search for a better structural fit. Two candidates have been pointed out: an *I*2/*m* (tilt a^0^b^–^b^–^) and an *R*-3 (tilt a^–^a^–^a^–^) SG. The latter was also reported for some other double perovskites, e.g., Ba_2_SrWO_6_: ICSD 246108, 246114. Applying any of those two space groups resulted in a noticeable improvement of agreement factors in comparison to cubic option (e.g., *R*_*wp*_ = 23.5% for *Fm*-3*m*, versus 20.9% for both *R*-3 and *I*2/*m* variants). Issues in distinguishing patterns of those two specific space groups have been mentioned in some papers (i.e., Zhou et al.)^59^ and resolved mostly basing on synchrotron and neutron diffraction experiments. Unfortunately, we had no opportunity to perform them. However, those are also the most plausible fits, considering other SGs and Glazer tilt systems supported by reported DFT calculations.^60,61^ Along with the other techniques used in this paper supported by some group theory calculations (see the following sections), one space group will fit more adequately later on.. For the sake of limiting the discussion lets assume for now it will be *I*2/*m*. The Rietveld refinement using this space group is depicted in Figure 3b with the following extracted unit-cell parameters: *a* = 6.0376(0) Å; *b* = 6.0369(0) Å; *c* = 8.5420(0) Å; β = 90.015(0)°; as one can notice, the monoclinic distortion is very small compared to the ideal cubic phase but still noticeable along uniquely distinguished 'b' axis. In the other *R*-3 variant, the lattice parameters are *a* = 6.0381(8) Å; *c* = 14.7905(0) Å. Its XRD diffraction pattern looks almost the same. All close-call matches alongside with other plausible SG models are available in Figure S3.

**Figure 4:**
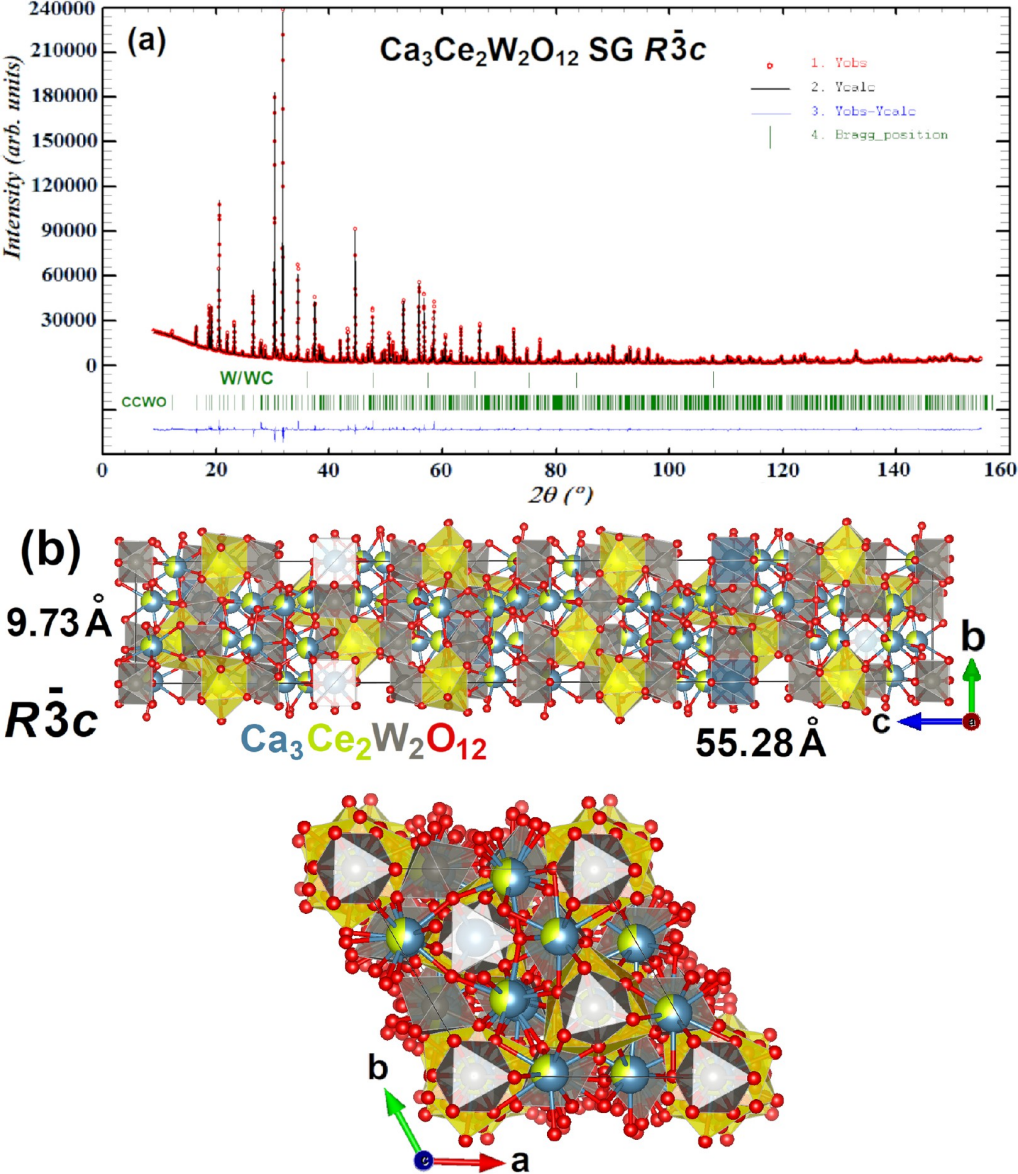
Original XRD pattern (a) after Rietveld fitting and refinement of the best, 99.7% pure *R*-3*c* phase containing only minor traces of W/WC impurities. Red dots are the manifestation of the real, observed pattern, the straight black line is the calculated fit, and the blue line depicts the difference between them. Green, vertical lines are specific Bragg positions assigned to specific phases. Unit cell in partial ball–stick, partial polyhedral filling convention is shown in (b) along “c” (down) and “a” (up) axes. One might notice that some sites are partially shared by both calcium and cerium atoms so only two of them (with different shapes and most pure occupation) were chosen to be depicted as polyhedral to maintain visibility.

Ca_3_Ce_2_W_2_O_12_’s ilmenite-like pattern in Figure 4a also shares an uncanny resemblance to its isostructural counterpart Ca_3_La_2_W_2_O_12_ JCPDS 49-0965,^62−64^ which in turn, was originally compared to Ca_5_RE_2_O_12_ by Villars et al.^65^ In that paper, only lattice constants of Ca_3_La_2_W_2_O_12_ were determined without a full structural refinement. Therefore, the current work presents, for the first time, a detailed structure description of Ca_3_RE_2_W_2_O_12_-type material after meticulous Rietveld processing in Table S1. Calcium and RE atoms share mostly the same sites with varying occupation factors (exhibiting so-called substitutional disorder), as was suggested by Li et al.^62^ Because of the Rietveld refinement, we were able to obtain the exact occupancies of our Ca/Ce sites in CCWO; they closely resemble Ca/La sites from the aforementioned articles. Here, however, SG assignment seems to be rather more straightforward; it is a nicely fitting rhombohedral *R*-3*c* (same as in Ca_5_RE_2_O_12_). But here the unit cell of CCWO is huge; it is composed of a staggering amount of 342 atoms as can be seen in Figure 4b. The lattice parameters are as follows: *a*, *b* = 9.7258(3) Å; *c* = 55.2793(0) Å. The noncentrosymmetric variant *R*3*c* (with *a*, *b* = 9.7258(2) Å; *c* = 55.2791(0) Å) is, however, indistinguishable by the powder XRD as they exhibit genuinely the same *hkl* powder patterns. Therefore, a lack of symmetry center cannot be rejected, and further studies are required just like in the case of BCW - please follow upcoming sections regarding Raman and group theory calculations for that sole purpose. On that basis, *R*-3*c* was chosen as a more probable, comparable, and safe variant to limit unnecessary discussion. All conventional reliability factors of refinement regarding acknowledged SG and their coexisting minor impurities are summarized in Table 2. Alternatives are presented the same way within Table S2. CIF related files regarding most plausible SG i.e. *I*2/*m* and *R*-3 for BCW, and *R*3*c* with *R*-3*c* for CCWO were respectively uploaded to CCDC database and attached to Supplementary files of this work.

**Table 2:** Conventional Rietveld Reliability Factors for Chosen BCW and CCWO SG and Their Diffractograms Hosting All Present Bragg Contribution Patterns Corrected with the Background

**Formula**	**SG**	**Z**	**V (Å^3^)**	**d_cal_(g/cm^3^)**	**R_B_**	**R_P_**	**R_WP_**	**R_EXP_**	**N_σ_ GoF**	**χ2**	**Fract (%)**
Ba_2_CeWO_6_	*I*2/*m*	2	311.342	7.410	13.5	23.6	20.9	4.36	1304.809	22.5	94.92
CeO_2-x_	*Fm*-3*m*	4	164.194	6.963	43.2						2.39
BaO	*Fm*-3*m*	4	168.010	6.062	36.7						2.37
W(/WC)	*Im*3̅*m*	2	31.695	19.264	98.9						0.32
Ca_3_Ce_2_W_2_O_12_	*R*-3*c*	18	4528.414	6.338	4.29	8.31	9.72	2.67	799.386	13.2	99.63
W(/WC)	*Im*3̅*m*	2	31.683	19.261	67.0						0.37

Some traces of impurities were detected, such as minor W(/WC) sinters ∼0.3% for both CCWO (∼99.7% pure) and BCW (batched ∼87–95% pure, best 95%). Low W(/WC) metal–ceramic remains, forming in both samples probably originate from sintering carbonate ingredients together with WO_2_ in a continuous-flow furnace devoided of air. Furthermore, because of slowly evaporating WO_2_ mixed with slow W(/WC) creation, some type of undetached ceria is also to be expected. Therefore, in BCW, the remaining minor peaks in Figure S4 were initially modeled to be a mixture of cubic BaO and CeO_2_ in a ∼1:1 ratio. However, the obtained lattice constants of CeO_2_ were so unexpectedly large (in comparison to ICSD 88759 reference) that the suggestion of some inexplicable distortions from free oxygen defects trying to bond between Ba–O–Ce were taken into account. Furthermore, after some synthesis attempts, a better explanation for those discrepancies was provided; when a configuration of CeO_2–*x*_ is assumed, i.e., Ce_7_O_12_, ICSD 4113 (see Figure S5), the Bragg match is indistinguishably close. Mixed cerium oxide might also appear as a side effect to the spontaneous charge transfer phenomena that we observed later during XPS, EPR, and (NUV)PL experiments described at sections 3.5 and 3.6. The widely assumed form of inflated cubic CeO_2–*x*_ could also be ascribed to Ce_7_O_12_ or more elusive Ce_3_O_5_ ICSD 621709 pattern.^66,67^ In our case, nearby, weak BaO reflexes make it difficult to ascertain. It might be an attempt of BaCeO_3_ formation. Nevertheless, both minor phases still form despite using an inert atmosphere, high pressure, and a slight 2–3% mass surplus of WO_2_. One must be aware that BaWO_4_ will form if additional WO_2_ content is too high; the remaining BaO reacts faster with lingering WO_2_ due to low kinetic barriers in synthesis activation process for scheelite.^68^ A strong peak around 26–28° characteristic to Ba or CaWO_4_ will then appear along with other, smaller satellites as can be seen in Figure S6. This is also the predominant phase if a solid-state reaction is performed in the air.

### 3.3 Raman and FTIR Spectroscopy

**Figure 5:**
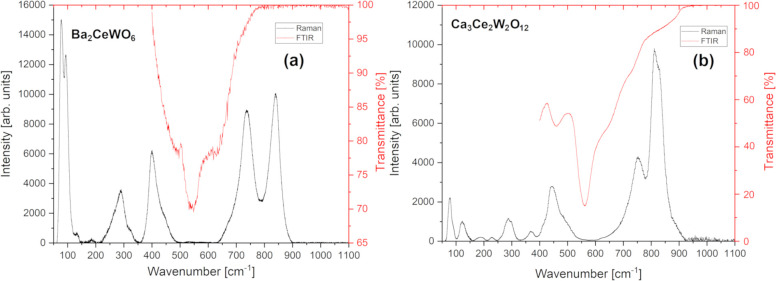
Raman (black) and FTIR (red) spectra of (a) BCW and (b) CCWO collected at ambient, room temperature conditions in the region of interest; no additional signals were detected above 1100 cm^–1^ (up to 4000 cm^–1^).

For both dark BCW and brownish CCWO, group theory calculations were performed according to the previously refined powder XRD data and extracted Wyckoff positions; irreducible representations for all four possible SG are gathered in Table S3. The aforementioned data were compared with experimental Raman spectra collected at ambient conditions and conveniently fitted in hope of finding a possible solution to the assigned SG controversy. For BCW it is *I*2/*m* vs *R*-3; and concerning CCWO, there is no distinction within the same Laue group, *R*3*c,* and *R*-3*c*. After processing all irreducible representations for each material one can observe that the experimental data collected at room temperature are similar to one another, and as a consequence, not many conclusions could be drawn at this point. Only a few trends could be distinguished: in BCW, *I*2/*m* and *R*-3 SGs host the same number of modes—12 in total. 7A_1g_+5B_g_ for the former group, and 4A_g_+4^1^E_g_+4^2^E_g_ for the latter. Both problematically fitting Raman spectra predicted in Figure 5a; for CCWO, *R*3*c* seems to meet the satisfactory, lesser number of Raman 7A_1_+14E phonons as shown in Figure 5b. Crude experimental positioning with brief characterization was enlisted and scientifically supported^69−74^ in Table 3. FTIR spectra have been proven to contribute less toward the whole discussion, so their literature-based interpretation^74−79^ and commentary were moved to Supporting Information p S11 with Table S4.

**Table 3:** Conjoined Experimental Data (Collected in Ambient Conditions Using a Confocal NIR-785 nm Laser) and Literature Phonon Assignments for Raman Spectra of Both BCW (Assumed *I*2/*m* or *R*-3) and CCWO (*R*-3*c* or R3c) Space Groups Emphasizing Observable Differences and Similarities^69−74^

**Wavenumberω_0_ (cm^–1^)**		**Signalassignmentandcharacteristics**^a^	
**Ba_2_CeWO_6_**	**Ca_3_Ce_2_W_2_O_12_**		
78.6	81.3	CaO_6_ and BaO_6_ multisite complex lattice deformations at the A-site of mixed B_g_/E_g_ and E origin signifying disorder and divergence from a perfect cubic structure	BCW s, sh
			CCWO m,sh
96.5	92,4		BCW s, sh
			CCWO mw, sh
134.1	123.5		BCW w, sh right shoulder
			CCWO mw, sh
182.8	186.3		BCW w, br
			CCWO w, br
231.2	228.3	in-plane σ_ρ_ and out-of-plane σ_τ_ bending in CeO_6_ and WO_6_ clusters of mixed B_g_/E_g_ and E origin between shared and not shared sites of various B-site cations	BCW, w, sh, left shoulder
			CCWO w, br
267.0	261.7		BCW mw, sh, left shoulder
			CCWO w, br, right shoulder
288.3	288.6		BCW m, sh
			CCWO mw, sh
320.3	297.7		BCW mw, sh, right shoulder
			CCWO mw, sh
	370.8	in-plane σ_sc_ and out-of-plane σ_ω_ bending in CeO_6_ and WO_6_ clusters of mixed B_g_/E_g_ and E origin between shared and not shared sites of various B-site cations	mw, sh
400.1	414.5		BCW m, sh
			CCWO mw, sh, left shoulder
441.9	445		BCW mw. sh, right shoulder
			CCWO m, sh
459.9		distorted impurity of ν_sym_ CeO_2_, (F_2g_)	mw, sh, right shoulder
	499.3	transverse CeO_6_ and WO_6_ motion of mixed A_1g_ and B_g_/E_g_ character between shared and not shared sites of various B-site cations	mw, sh, right shoulder
	626.7		w br, right shoulder
667.2-	672.4		BCW mw, sh, left shoulder
			CCWO w, br, right shoulder
	710.8	ν_asym_ and ν_sym_ stretches of CeO_6_ and WO_6_ clusters of A_1_ and A_1g_ for various Wyckoff sites not only partially occupied but also hosting B-site Ce/W ions having different charges	mw, sh, left shoulder
733.5	753.2		BCW s, sh
			CCWO m, sh
814.5	812.8		BCW m, sh, left shoulder
			CCWO s, sh
838.9	828.3		BCW s, sh
			CCWO s, sh
	888.1		mw, sh, right shoulder

a Key: w, weak; m, medium; s, strong; sh, sharp; br, broad; ν_sym_, symmetric stretching; ν_asym_, asymmetric stretching; σ_sc_, scissoring deformation; σ_ω_, wagging deformation; σ_τ_, twisting deformation; σ_ρ_, rocking deformation.

However, one must take into account several other factors and literature^80−85^ before passing any final verdict about those assessments and moving on toward nonambient measurements. First, note that all of pinpointed Raman peaks are really broad; their fwhm is often in between 36 and 50 cm^–1^. This might not only suggest some degree of disorder or internal vacancies present within discussed materials but, such a large convolution of neighboring bands could also accommodate for different charges of BB'-site ions. It would be relatively hard to distinguish between satellites properly without any high resolution or polarization measurements supported by precise DFT calculations. Furthermore, since currently selected, default SGs are tilted and not as simple as widely discussed *Fm*-3*m* hosting 3d M^2+^-W^6+^5d ions, additional signals of miscellaneous origin would be expected here. This is especially true for very weak, spontaneously appearing peaks like those (of 300–400 r.u. intensity or less) between 540 and 590 cm^–1^, barely distinguishable from the noise. Their true presence is debatable, but if true their assignment could fit one of nontrivial E_g_ (*R*-3) or rather B_g_ (*I*2/*m*) modes denoted as difficult to spot in the respective literature.^69,85^

Second, most batches of BCW possess an unusual yet strong Raman feature at ambient conditions, near 460 cm^–1^. It can be crudely assigned to the remaining CeO_2_ (splitting F_2g_ mode) lingering due to slow evaporation of WO_2_ which is simultaneously oxidizing and bonding with excessive BaO during the final stages of synthesis; Figure S7 is consistent with such a scenario. But, as shown earlier by XRD and later XAS measurements, this highly distorted CeO_2_ (inflated most likely by the presence of Ce^3+^) could be Ce_7_O_12_. These kinds of ceria have additional, weak but broad signals near 230–250 and 550–600 cm^–1^ from interstitial defects and lattice deformations.^86−88^ This additionally complicates the interpretation of presented spectra, especially in the latter range where genuine DP signals could emerge like in our case.

Third, the low power of applied laser radiation was also an important factor during such long exposition times since both samples are exceptionally heat-sensitive in the air and their dark coloring contributes greatly to decomposition via dark-body absorption. These effects are visibly proven by the yellowish or rather white AWO_4_ hue appearing on the pellet’s surface depicted previously in Figure 2b or S2. Measurements of those discolored edges, in BCW, revealed such characteristic peaks, marked as black asterisks in Figure S7 Raman spectra. They nicely match our pure barium tungstate reference.^9^ Knowing that non-radiative energy dissipation impairs the shape and form of actual results via gradual oxidation one should mainly consider using NIR lasers to avoid local overheating, especially if performing heat-dependable measurements without any inert, protective atmosphere.

**Figure 6:**
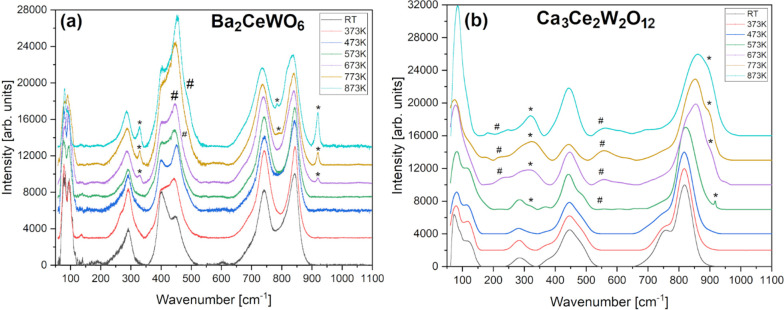
Raman spectra for (a) BCW and (b) CCWO collected in the function of heating from room temperature up to 873 K in air (without protective, inert N_2_ atmosphere) showing noticeable features of decomposition mostly above 573 K. Asterisks mark modes originating from forming tungstates (A_*x*_WO_4_), and hashtags signify a group of various, detached CeO_2–*x*_ oxides.

In light of the foregoing facts, careful, spectral scattering measurements outside of ambient conditions were needed, namely at low as well as high temperatures and pressures. During intentional heating in air, as depicted in Figure 6, some changes of the Raman spectra started to develop already between 573 and 673 K due to degradation. Besides steady blue-shifting, the intensity of a few bands visibly changed because cerium bonds reform at these conditions by separating from the original matrix. Hence, CeO_2–*x*_ modes strengthen or generally appear in both materials with a few sharp-but-weak peaks associated with tungstate's separation. The latter signals further grow, up to 773–873 K which is the functional threshold of the HT apparatus. Just at that point, W^4/5+^ ions (if present–mostly in BCW sample) visibly oxidize forming a family of compounds gradually morphing toward A^2+^W^6+^O_*x*_ scheelites. Meanwhile, the CCWO modes swing back and forth moving slowly to higher wavenumbers most likely because of the separation of 3+ ceria from the original matrix. In an inert, N_2_ atmosphere both compounds are safe from oxidation. Still, no crucial changes, in form of second-order phase transition toward higher order cubic SG were observed, as shown in Figure S8. Just a recoverable blue shift of wavenumber position vs temperature was noted with slow amorphization visualized by a gradual broadening of several peaks during cool down.

**Figure 7:**
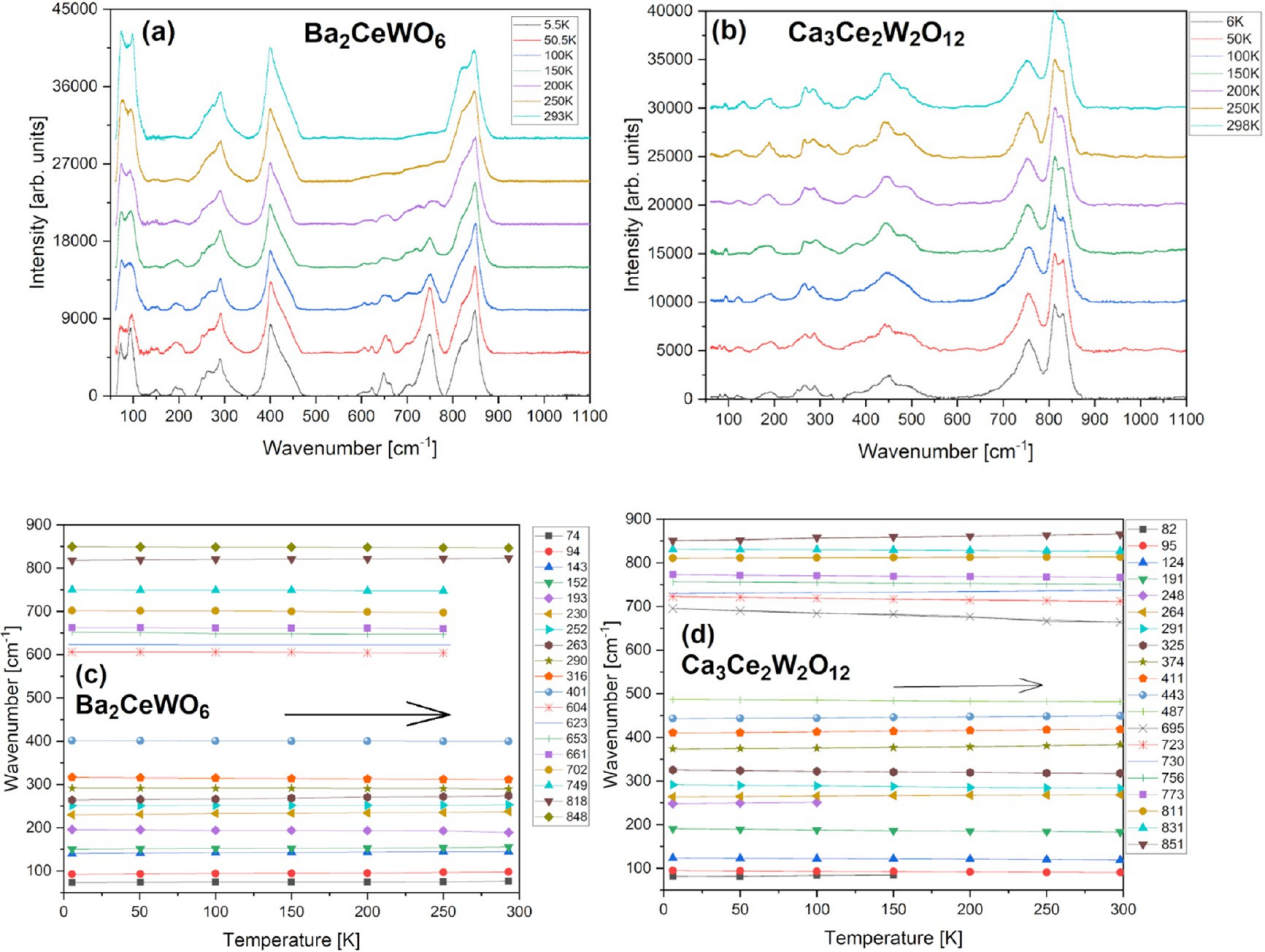
Raman spectra for BCW (a) and CCWO (b) collected as a function of heating from 5 K to room temperature in a high vacuum after thorough He gas flushing. Peak positions vs temperature (symbols) are depicted for both BCW (c) and CCWO (d), respectively. Solid lines are linear fits.

Measurements at cryogenic conditions could resolve some critical issues in terms of SG discussion. Indeed matching BCW’s *I*2*/m* SG with *R*-3 makes much more sense in cryogenic conditions. In Figure 7a we can see much more, previously invisible or weak (6) modes in the middle of the spectra (at 600-700 cm^–1^). Peaks appear just below 200 K and, at around 100 K, they rapidly deconvolute and sharpen while cooling toward helium-level temperatures (∼5 K). This might suggest a broad-range *R*-3⇄*I*2*/m* second-order phase transition in subzero temperatures with some partial coexistence of both phases in a metastable state at room conditions. This scenario was previously discussed by Lufaso et al. in his high-order DP paper;^89^ however, the transition transpired at much higher temperatures, around 373–400 K. Thus, the discussed case of two competing, *I*2/*m* and *R*-3 SG with initially declared 12 modes (mentioned at the beginning of this section, in Table 3) still remains unanswered. We would need to substract at least 6 peaks to fit the required, theoretical number stated in Table S3. There are at least 18, suggesting that this could be happening due to: RE cationic Ce/W BB′-multisite charge-splitting; some apparent tilting effect; or eventual reemerging interstitial oxygen defects. Yet still the behaviorism of the sample seems uncanny, and the issue is still unresolved without any evidence from LT powder XRD measurement. More plausible outcome would be a sharp first-order phase transition from *I*2*/m* to *P*2_1_/*c* SG. That would account for all those aforesaid, additional signals.

Cooling CCWO results in much more subtle, if any, changes as shown in Figure 7b Raman spectra. To the best of our knowledge, no outstanding phase transition occurred within that substance. Only simple, linear wavenumber vs temperature dependencies of all modes were observed as visualized in Table 4. If we take into account all original, ambient peaks, which were gradually sharpening during cooling, even BCW does not show many deviations from the linear fittings. That is why we are skeptical about the origin of any self-declared transformation at that point. Considering the similarity between both XRD patterns for any of those two specific SG, highly sophisticated XRD or rather neutron measurements are required at LT to pass any final verdict on such claims.

**Table 4:** Linear Fit Parameters Obtained for Temperature Dependent Data Presented in Figure 7 for Both Investigated Materials^a^

**Ba_2_CeWO_6_**			**Ca_3_Ce_2_W_2_O_12_**		
**ω (cm^–1^)**	**dω/dT(cm^–1^/K)**	**R^2^**	**ω (cm^–1^)**	**dω/dT(cm^–1^/K)**	**R^2^**
74	0.0095 ± 0.0025	0.69	82	0.0202 ± 0.0064	0.83
94	0.0177 ± 0.0015	0.96	95	–0.0150 ± 0.0010	0.98
143	0.0163 ± 0.0017	0.94	124	–0.0137 ± 0.0015	0.94
152	0.0171 ± 0.0022	0.91			
193	–0.0186 ± 0.0041	0.77	191	–0.0254 ± 0.0017	0.98
203	0.0230 ± 0.0021	0.95	248	0.0286 ± 0.0029	0.99
252	0.0056 ± 0.0012	0.78	291	–0.0284 ± 0.0018	0.98
263	0.0346 ± 0.0018	0.98	374	0.0325 ± 0.0034	0.95
290	–0.0035 ± 0.0009	0.71	411	0.0288 ± 0.0014	0.99
316	–0.0179 ± 0.0007	0.99	443	0.0224 ± 0.0017	0.97
401	–0.0051 ± 0.0008	0.87	487	–0.0211 ± 0.0020	0.96
604	–0.0139 ± 0.0024	0.84	695	–0.1080 ± 0.0084	0.97
653	–0.0200 ± 0.0033	0.86	723	–0.0381 ± 0.0025	0.98
661	–0.0190 ± 0.0045	0.73	730	0.0254 ± 0.0014	0.98
702	–0.0199 ± 0.0023	0.92	756	–0.0194 ± 0.0010	0.97
749	–0.0086 ± 0.0015	0.83	811	0.0083 ± 0.0011	0.92
818	0.0158 ± 0.0009	0.98	831	–0.0135 ± 0.0011	0.97
848	–0.0095 ± 0.0009	0.94	851	0.0507 ± 0.0047	0.95

a Several deconvoluted lines slightly differ from the ambient temperature line positions ω_0_ in Table 3 probably due to lattice relaxation and post-experimental conditions in cryogenic equipments. The increased number of BCW bands at LT, almost matching those of CCWO, suggests a phase transition.

**Figure 8:**
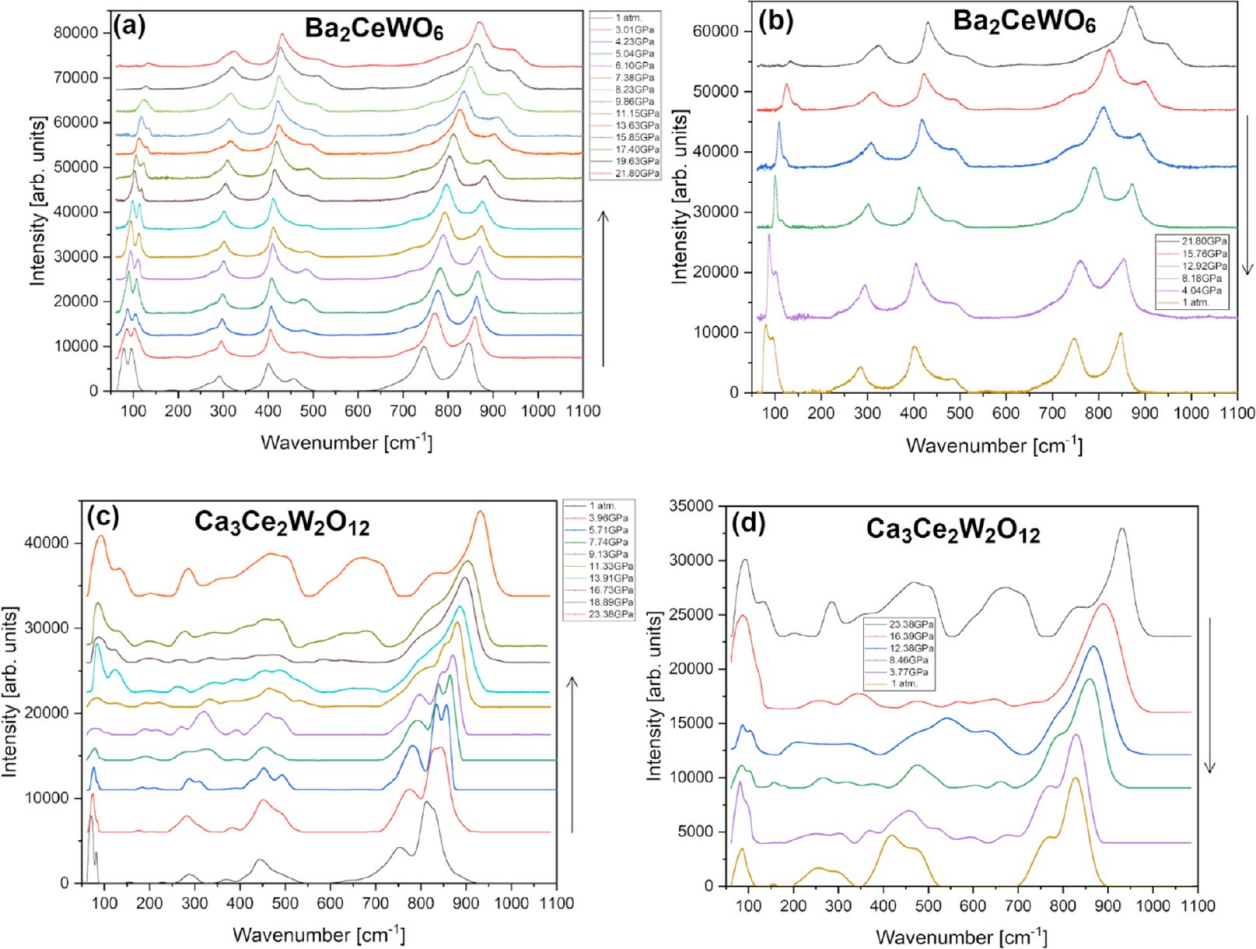
Raman spectra collected under hydrostatic argon PTM while using consecutive compression–decompression cycles (left-to-right) for: BCW (a,b) and CCWO (c,d). Arrows guide the eye. Despite apparent, slow amorphization accompanied by red-shifting peak positions some interesting, new, nonlinear changes could be only noted for CCWO above 9 GPa.

**Figure 9:**
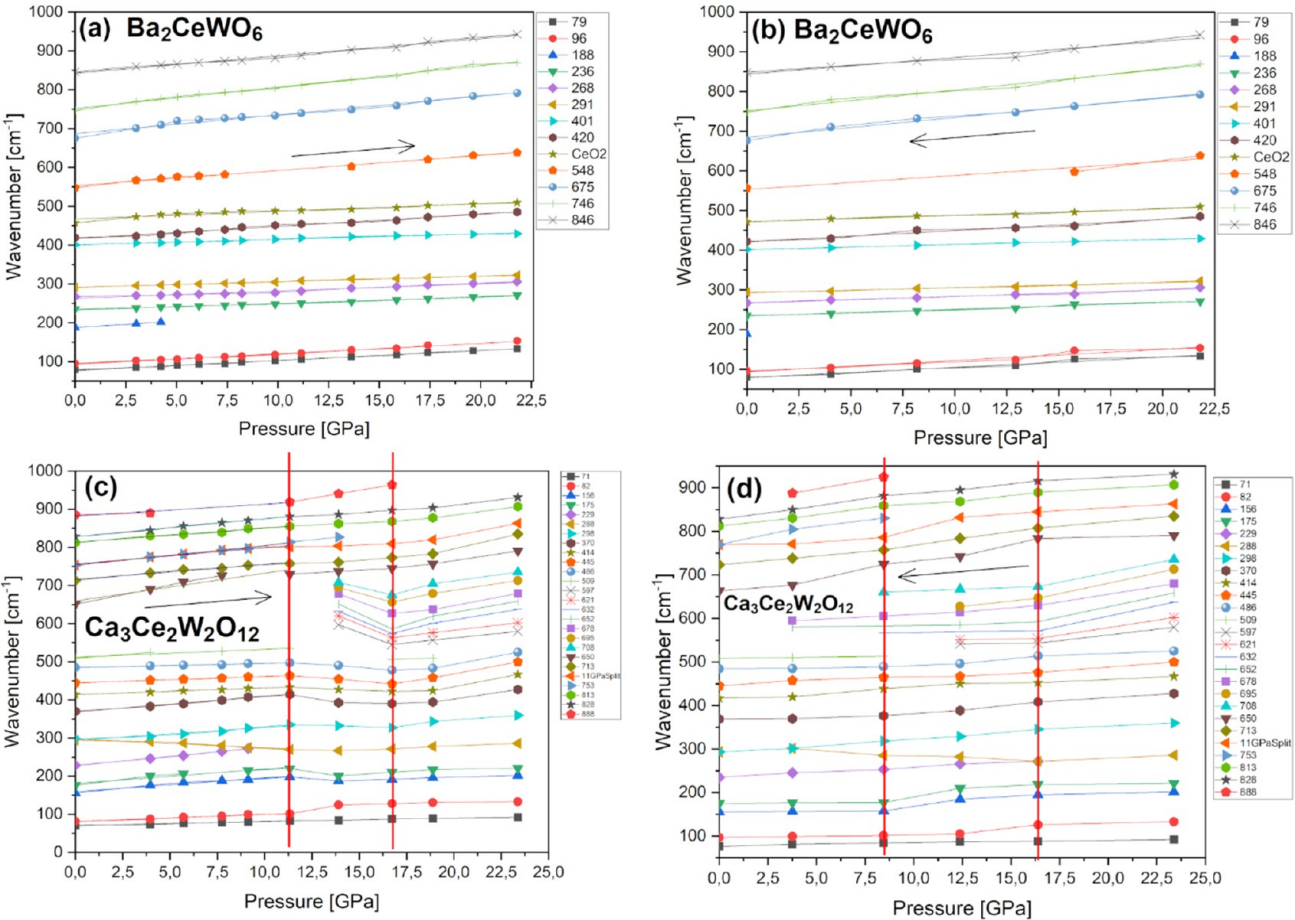
Raman peak position vs pressure relations for modes showcased in previous Figure 8. Compression–decompression studies are shown in the same, respective order for BCW (a,b) and CCWO (c,d). Inserted right and leftrrows guide the eye. Red vertical lines in (c,d) cut the pressure range into 3 sections to emphasize the transpiring changes in CCWO.

According to group theory analysis, matching CCWO structure with the centrosymmetric *R-*3*c* SG solely with room temperature data is not good since it does not account for several, extra Raman modes — +6A_1_ and +13E that would be needed unless they are all degenerate. But if one will look into the results of high-pressure measurements not everything seems to be as clear. Raman spectra measured under increasing and decreasing pressure are shown in Figure 8ab for BCW and Figure 8cd for CCWO, respectively. The pressure dependencies of peak positions are presented in the same fashion in Figure 9, while the determined pressure coefficients are gathered in Table 5. As can be seen in Figure 8a, nothing unusual happens in terms of BCW polymorphism and eventual phase transitions; only moderate amorphization and peak intensity decay were noticed during pressurization. This is an argument against SG *I*2*/m* assignment since behavior claimed by Howard et al. suggests otherwise - *R*-3 would rather match that statement as depicted in modified Figure S9.^90,91^ However, this sample might not have reached the transition point just yet. Moreover, as peaks red-shift linearly while slowly decaying and broadening during compression in Figures 8a and 9a, it makes such assessment even more difficult. Especially in terms of establishing the exact number of distinguishable modes for a typical *I*2/*m* structure (originally having ∼12 modes). In addition, only slight hysteresis occurs around 8–9 GPa (not bigger than 2 GPa) while releasing pressure, as can be observed in Figures 8b and 9b. If compressed only to or just below that point, peak vs pressure dependencies recover identically. This means that only partial amorphization could explain such phenomena, but, to pass fair judgment high-pressure XRD would also be required in the future.

**Table 5:** Room Temperature Wavenumbers and Pressure Coefficients of Raman Lines Presented Previously in Figure 9 for Both BCW and CCWO^a^

**Ba_2_CeWO_6_**			**Ca_3_Ce_2_W_2_O_12_**		
**ω (cm^–1^)**	**dω/dP to 22 GPa(cm^–1^/GPa)**	**R^2^**	**ω (cm^–1^)**	**dω/dP to 9 GPa(cm^–1^/GPa)**	**R^2^**
79	2.570 ± 0.038	0.99	71	1.089 ± 0.078	0.98
96	2.662 ± 0.067	0.99	82	1.853 ± 0.124	0.98
			156	3.614 ± 0.372	0.95
188	3.268 ± 0.171	0.99	175	3.781 ± 0.584	0.91
236	1.649 ± 0.051	0.98	229	4.810 ± 0.146	0.99
268	1.875 ± 0.097	0.96	288	–2.286 ± 0.312	0.91
291	1.452 ± 0.027	0.99	298	3.348 ± 0.381	0.94
			370	4.019 ± 0.188	0.99
401	1.405 ± 0.046	0.98	414	1.755 ± 0.064	0.99
420	3.155 ± 0.105	0.98	445	1.681 ± 0.053	0.99
			486	1.029 ± 0.060	0.9̀8
548	3.903 ± 0.128	0.99	509	2.075 ± 0.407	0.89
675	4.825 ± 0.218	0.97	650	7.266 ± 1.369	0.90
			713	3.915 ± 0.329	0.97
			Split	4.327 ± 0.363	0.97
746	5.523 ± 0.137	0.99	753	5.133 ± 0.223	0.99
			813	3.704 ± 0.230	0.98
846	4.453 ± 0.155	0.98	828	4.703 ± 0.126	0.99
			888	3.089 ± 0.707	0.95

a In CCWO linear pressure coefficients were determined only for the pressure range up to 9 GPa, after which significant changes in the line positions were noted indicating a possible phase transition.

In contrast to BCW, rhombohedral CCWO is considered to be a much more disordered ilmenite, so its structural behavior should be even more erratic and unpredictable. As Figure 8c shows, that statement is entirely true. Its validity is settled as much stronger amorphization occurs (by increasing band width) while a more diverse shifting gradient transpires among all the peaks, up toward 9 GPa. Furthermore, in Figure 9c, above 9 GPa a set of new, metastable peaks appear around 550–650 cm^–1^, and some of them swing incoherently around their original positions while rapidly raising their intensity for the next +4–5 GPa. This situation stabilizes around 16 GPa, but conclusions are still uncertain regarding to which exact phase this compound changes into — the total number of peaks and group theory suggest some type of monoclinic second-order phase transition (maybe to monoclinic *C*2/*c*) but precise, experimental assignment, with high-pressure powder XRD measurements would be required. A similar situation, in the matter of precedence, was noticed in precursor BaWO_4_ codoped with Ce^3+^ and Na^+^ which encountered a metastable fergusonite (*I*2/*a*) phase between 7 and 9 GPa before transferring also into monoclinic *P*2_1_*/n* SG.^9^ However, considering contradictory behavior of the isostructural BCW (assuming it has *R*-3 SG), if truly no phase transition occurs at that period, the higher count of modes in CCWO would be a plausible match for *R*-3*c* pick, especially if those spontaneous, close-to-noise artifacts near 550–580 cm^–1^ are real at ambient conditions as stated in the literature.^90,91^ Aforementioned SG could transition to the monoclinic phase via a similar route as *R*-3 does to *I*2/*m* (*C*2/*m*) but with different stimuli. This would also explain the extensive number of registered peaks (+20 at best but some of them might be degenerate), especially if different cations occupy and share various A/B-site Wyckoff positions, like in our case partially substituted Ca/Ce with different Ce–W charge couplings. Decompression, presented in Figure 8d and Figure 9d, clearly shows much bigger hysteresis (±4 GPa) and amorphization persisting at least twice as long as in BCW. Some of those aforementioned, newly acquired peaks still remain below the original transition region, although significantly weaker. Given these facts, one can not be really sure whether those peaks are just emerging defects (like BB′-site splitting modes of various Ce–W ion pairs losing their bonding strength with oxygen due to released pressure and lessening tilting) or just a fingerprint of a genuine phase transition. Conventionally unavailable high-pressure neutron measurements would resolve such issues.

### 3.4 High-Temperature Studies: DSC, TG, and XRD

**Figure 10:**
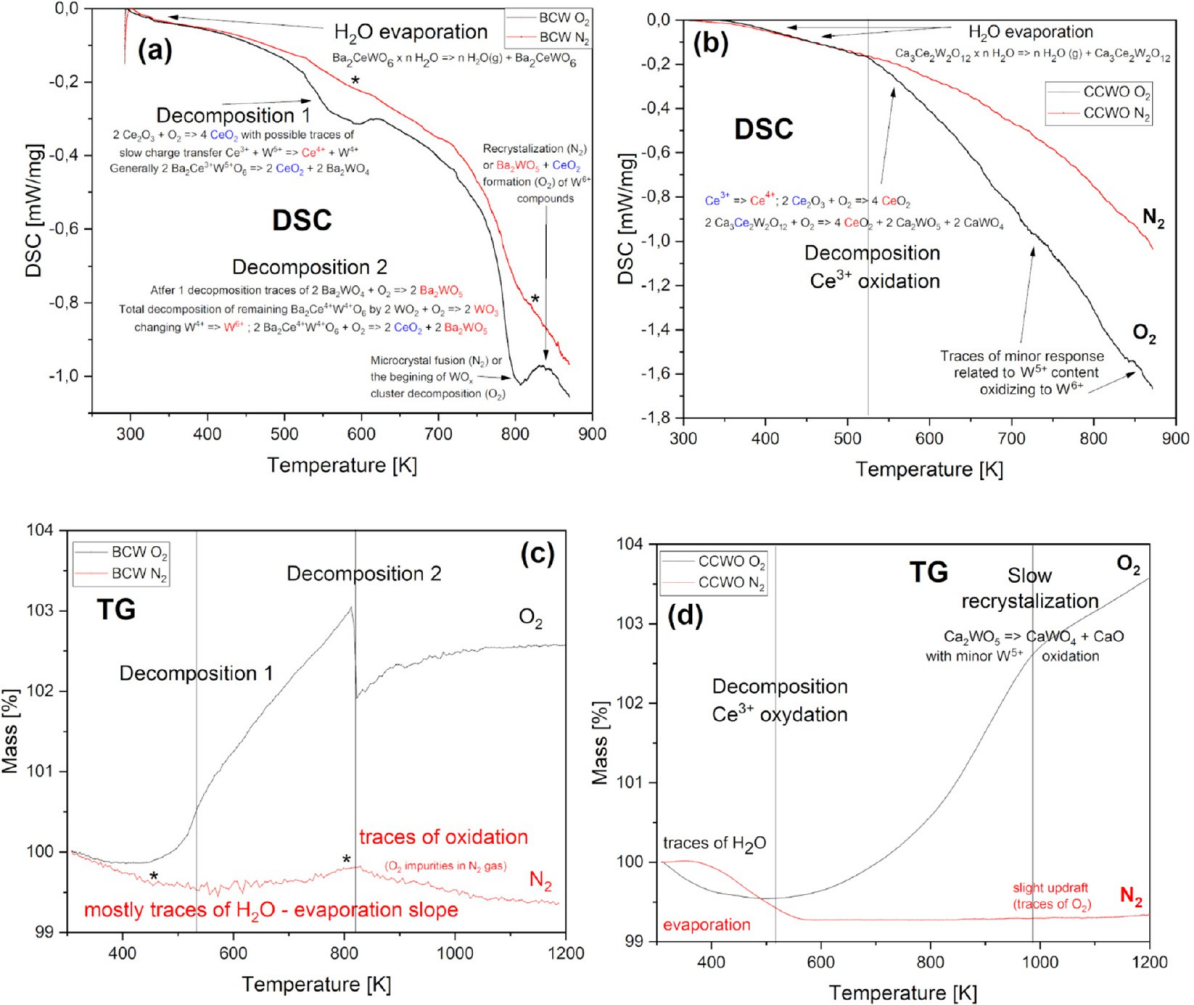
Differential scanning calorimetry (top) and thermogravimetry (bottom) scans of Ba_2_CeWO_6_ (a,c) and Ca_3_Ce_2_W_2_O_12_ (b,d) measured in air (black lines) and inert N_2_ gas (red) atmospheres. Asterisks in (a,c) mark the temperatures at which oxygen is released from the matrix. Vertical lines separate regions in which different decomposition/oxidation processes occur, as described in the figure.

The results of DSC (a,b) and TG (c,d) measurements performed in N_2_ atmosphere, depicted by red lines in Figure 10, do not show much change in the region of interest–minor fluctuations were to be expected due to evaporation of intercalated water:
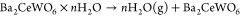
1
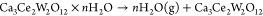
2

These results can serve as stability reference measurements for later heat capacity calculations. When exposed to elevated temperatures in the air (black lines), both BCW (Figure 10a,c) and CCWO (Figure 10b,d) show one or two stages of degradation, respectively. The first stage, around 573–623 K, is mainly attributed to Ce^3+^ oxidation according to the available literature.^92,93^ The second degradation period, above 773 K, is present mainly for BCW since only it has a large amount of W^4+^ ions prone to fast oxidation toward W^5+/6+^ which are the main ingredients of CCWO at that point.^94−96^ Unstable WO_3–*x*_ species containing 5+ ions have a higher heat-tolerance factor but will also start oxidizing since their decomposition barrier does not fall much above that threshold, though at a much slower pace.^53,95,96^ Hence, those continuous, downward slopes in DSC and TG graphs (Figure 10b,d) experience slight fluctuations and changes in their curvature above 900 K—possible, minor Ce^4+^/W^5+^ phases might already form by then.^97^

The respective chemical reactions regarding the aforementioned phenomena are presented below. They are not only partially supported by high-temperature Raman spectra (Figure 6 in the previous section) but also HT powder XRD diffraction patterns presented in Figure S10. As a brief commentary, it is worth noting that in the latter measurements fluctuations of peak positions and the disappearance (of old) or emergence (of new) signals at specific Bragg angles (while heating), prove that slow amorphization and decomposition proceed in both materials toward these specific products:
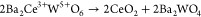
3
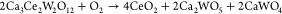
4

CCWO and a minor part of BCW experience decomposition via Ce^3+^ → Ce^4+^ oxidation at ∼575 K which transpires ideally as 2 Ce_2_O_3_ + O_2_ → 4 CeO_2_ reaction. An enclosed, oxygen-depleted environment might also result in the formation of some distorted, partially oxidized CeO_2–*x*_ species like Ce_7_O_12_ or, less likely, Ce_3_O_5_ but just on the pellet’s surface. This strictly depends on the amount of the oxygen stored within initially used reactants. BCW decomposition via *W*^*4/5*+^ → *W*^*6*+^ oxidation at *+773 K* ideally takes the form of 2 WO_2_ + O_2_ → 2 WO_3_ reaction but already present minor phases from the cerium decomposition stage could also react:
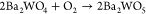
5
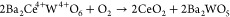
6

Further pyrolysis, at higher temperatures (beyond *in situ* X-ray, DSC, and TG equipment capabilities), pushes W^5+^ ions toward W^6+^ and restructures byproducts depending on available oxygen content.^98^ Frankly speaking, reaching II and III stage synthesis temperatures in the air is fatal as demonstrated by the post treatment (not *in situ*) measurements shown in Figure S11. Final products slowly turn out to be unambiguous Ba- or CaWO_4_ tungstates through the following reactions:
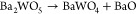
7
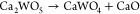
8

These reactions could account for occasionally encountered (in XRD) A^2+^ oxides. Both BaO and CaO, bizarrely do not seem to perfectly fuse with the remaining, excessive CeO_2-x_ content. They also do not react with moisture or natural CO_2_. This is happening probably due to the high dispersion rate of all oxides and ceria’s low reactivity.^96^

**Figure 11:**
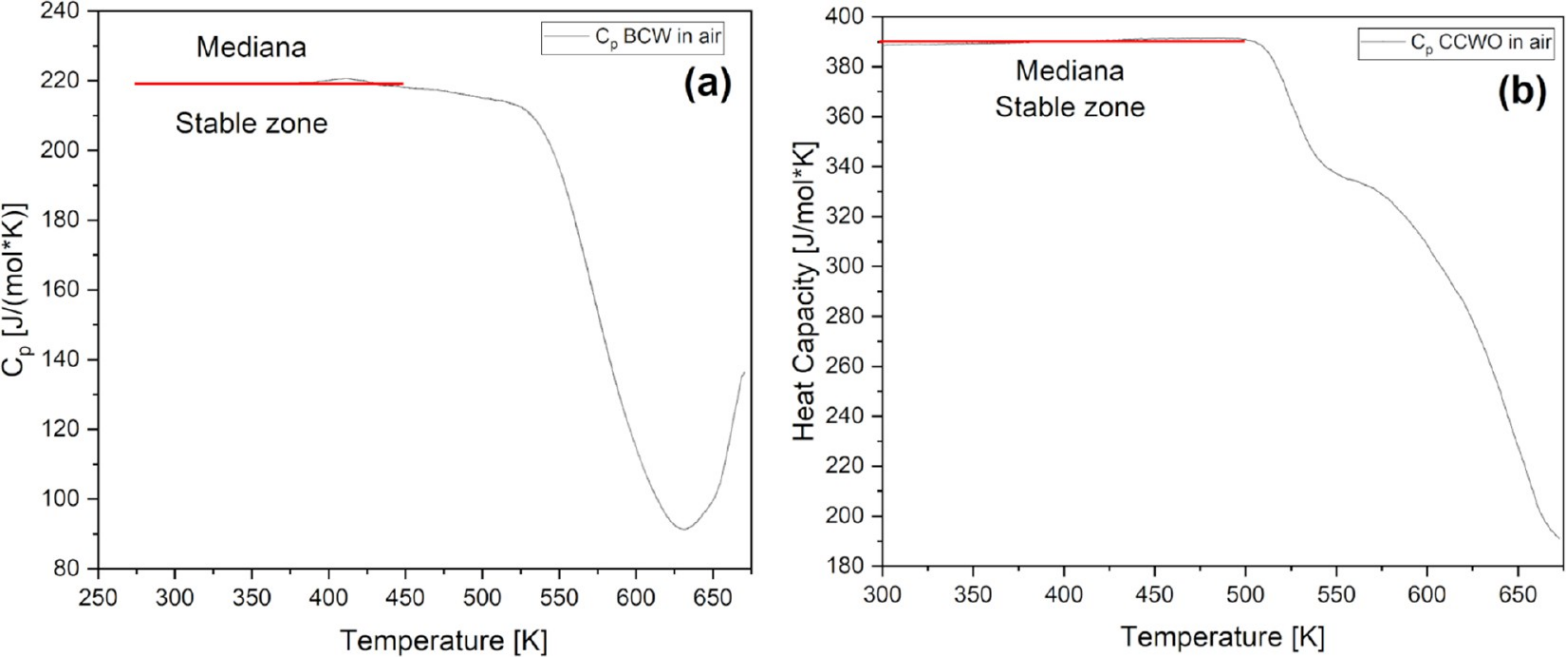
Molar heat capacity for (a) BCW and (b) CCWO compounds in the function of temperature derived from DSC measurements in air. The values given in the text refer to the stable zone marked by the red line.

Subsequently, research and calculations of heat capacity coefficients (Cp) were performed on dehydrated materials only to approximately 500 K where both seemed to be relatively stable. Heat capacity data, presented in Figure 11, were collected against sapphire reference considering future applications in the ambient atmosphere. For BCW, the average Cp value is ∼219.25 ± 0.52 J/(mol × K) while for CCWO it is ∼390.22 ± 0.87 J/(mol × K). Medians are equal to 219.05 J/(mol × K) and 390.24 J/(mol × K), respectively. These are still within acceptable levels considering materials of similar origin at room conditions.^99−101^ Concerning previously mentioned HT XRD studies, axial and volumetric expansion coefficients were also extracted, alongside angular dependencies presented in Figures S12 and S13 for BCW and CCWO, respectively. Linear fits suggest match-up to such heating expansion coefficient as suggested in Table 6.

**Table 6:** Crystallographic Expansion Coefficients for Ba_2_CeWO_6_ and Ca_3_Ce_2_W_2_O_12_ Deducted from Powder XRD Data in the Stable Region below 500 K

**Sample**	**Unit cell axis**			**Angle (α)**	**Volume(V)**
	**da/dT (Å/K ×10^–5^)**	**db/dT (Å/K ×10^–5^)**	**dc/dT (Å/K ×10^–5^)**	**dα/dT (deg/K ×10^–4^)**	**dV/dT(Å^3^/K ×10^–2^**)
BCW	9.83(0) ± 0.14(0)	8.36(9) ± 0.18(5)	10.48(7) ± 0.28(6)	–2.79(8) ± 0.17(7)	1.28(2) ± 0.18(6)
CCWO	14.19(6) ± 0.29(9)		78.63(1) ± 2.17(7)		19.94(1) ± 0.47(2)

### 3.5 XAS and XPS Data

The XPS surface analysis was mainly focused on determining whether or not Ce and W coexist within the same phase in a complementary, dualistic charge form since the registered binding energy of each given element’s electron orbital tells us about its specific ionic state. One can also conveniently calculate what is the total atomic content and the mutual ratio of the ions to estimate and verify the general chemical formula provided by the XRD database. The binding energy of electron orbitals for other divalent elements such as Ba, Ca, and O were additionally discussed here for that sole purpose; however, the complex nature of our materials turned out to have some interesting impact on other, more convoluted interactions. Since our powders were bad conductors XPS was performed on samples placed on a carbon tape. XAS was used first on not-grinded pellets as an alternative, in-depth supporting technique to ensure a proper qualitative assessment.

**Figure 12:**
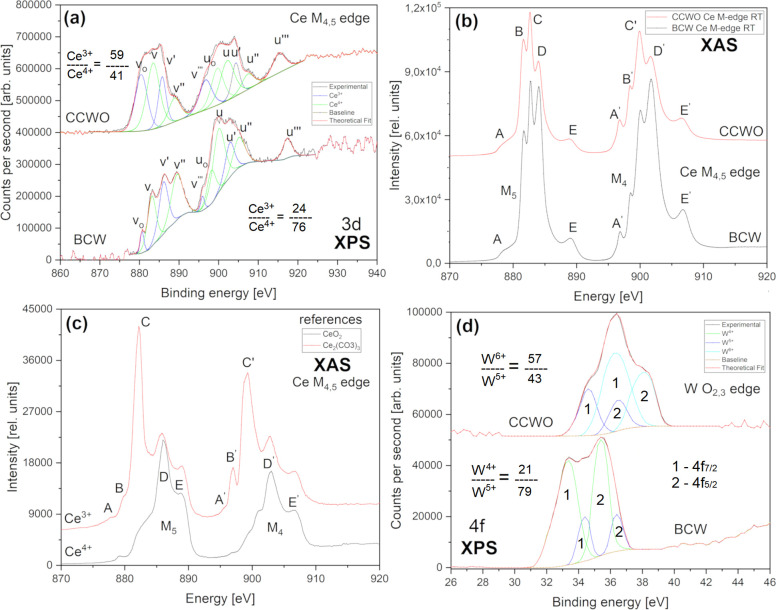
Crucial X-ray spectroscopy analysis collected at room temperature and ultrahigh vacuum for B-site cations, Ce and W, present inside our powders and pellets showing the mixed, dualistic nature of both materials. The bottom curves mostly feature BCW and the top ones CCWO. Cerium 3d measurements for both surface-sensitive (a) XPS and more penetrating M-edge (b) XAS methods are presented as two top graphs for the respective samples. (c) has XAS spectra of pure 99+% references used for all syntheses. (d) presents the only available XPS 4f_7/2_ and f_5/2_ results regarding tungsten’s charge state doublets. XAS W edges were, unfortunately, out of beamline energy range.

Cerium 3d spin–orbit multiplet binding energy and M_4,5_ absorption edges in Figure 12 show the complex nature and versatility of ionic forms present inside our compounds. BCW and CCWO XPS Ce 3d orbitals above 880 eV could be crudely deconvoluted into two sets (3d_5/2_ and d_3/2_) of five (v and u) peaks conjoined strictly together in Figure 12a. The spectrum of pure CeO_2_ (only Ce^4+^ ions) originally consists of three multiplets related to interactions with 4f^*n*^ (*n* = 0, 1, and 2) electrons denoted as v, v″, with v‴ (in the d_5/2_ component); and u, u″ with u‴ (d_3/2_ component) separated by 18.5 eV gap with the typical intensity relation v/u = 1.5 as shown by Romeo et al.^102−106^ We can also see peaks assigned to Ce_2_O_3_ (intentionally Ce^3+^ ions) which should exhibit two multiplets related to 4f^*n*^ (*n* = 1,2) denoted to v_o_, v′ as d_5/2_ components and u_o_, u′, respectively, as d_3/2_ bands. Peaks denoted as v_o_ and v′ are located in these exact reference positions and can serve as an evidence for self-occurring charge transfer phenomena present inside our compounds during HT synthesis.^107−110^ They represent about 24% of ceria in BCW and, analogically around 41% regarding CCWO. These ions were not intentionally added to the former compound and are most likely scattered randomly throughout the BCW matrix, being mixed in ∼1:3 ratio with their dominant Ce^4+^ counterpart. Their existence would support the creation of disrupted CeO_2–*x*_ impurities hosting most probably 3+ and 4+ cations in the Ce_7_O_12_ form. A similarly complicated multiplet of Ce M_4,5_ edges was observed in Figure 12b. Since the XAS spectrum is created by energy absorption and transition of deeper, much more penetrating 3d electrons toward empty 4f states (localized above Fermi level) in a larger volume, we could gather complementary bulk information from our investigated pellets. Thus, in our case, low energy peaks labeled A, B, and C for the M_5_ edge and A′, B′, and C′ for the M_4_ edge are characteristic of Ce^3+^ structures. Those labeled further as D (and D′) and E (E′) are assigned mainly to Ce^4+^ ions from the literature^111^ and reference spectra gathered by us from +99% pure substrates in Figure 12c. For now, much more pronounced peaks, D and E in BCW, confirm XPS findings of the compound’s predominant fraction of Ce^4+^ within Ba_2_Ce^3/4+^W^5/4+^O_6_ mixed neighborhood.^111−113^ Worth noting is that even our references show some dualism in highly scattered surroundings; two other major bonding energies from Ce^3+^ and Ce^4+^ ions (especially between B,D–B′,D′), suggest that some cationic impurities (having different charges) in large disproportion to those ratios encountered in our samples (in comparison to BCW) might still be at play. That probably originates from RE-oxides unstable, chemical character which seeks some sort of natural balance after synthesis while time passes by.^114^ Qualitatively speaking, these bands might analogically inform us about the future nature of our compounds, mainly 5d^2^4f^0^ → 5d^1^4f^1^ exchange splitting between Ce^4+^ predominant form (joined presumably with W^4+^) toward minor Ce^3+^ (being possibly shared with just as unstable W^5+^ pair).

BCW double-layered BaCeO_3_ × BaWO_3_ perovskite shares recognizable, but very different, oxygen corner arrangements in between unevenly scattered, interchanging ions, i.e., −Ba–O-Ce^3/4+^–O–Ba-O-W^5/4+^-O-. This might lead to four unique types of possible hybridizations between B-site ions with additional formation of vacancies. But, because some of them are situated in specific, lone occupation sites, the total valency of +8 must still somehow be maintained in order to sustain proper structural balance inside such ordered unit cell. Therefore, a specific cerium ion should have its complementary charge-equivalent counterpart localized elsewhere, in that case, at nearby tungsten. That is why the next logical step would be to pursue information about W 4f orbitals. Unfortunately, tungsten was out of the synchrotron’s beamline energy range so we must have relayed solely on XPS data shown in Figure 12d. There, one can clearly see two sets of partially overlapping 4f_7/2_ and 4f_5/2_ spin–orbit doublets per sample. The BCW diagram on the bottom has a lower energy doublet settled at a binding energy of 4f_7/2_ line 33.4 eV, a bit higher than for the original W^4+^ reference resembling WO_2_; the binding energy of ideal W^4+^ is reported to be roughly 32.9 eV.^115^ Together with a 4f_5/2_ peak (35.4 eV) they comprise approximately 79% of the line’s content. A higher doublet is close to W^5+^ but also not quite matches the referral positions — these WO_3–*x*_ lines have a binding energy of 4f_7/2_ near 34.4 eV and together with 4f_5/2_ (36.4 eV) consist of the remaining 21% W in BCW. Since there is little to no evidence of pure W^5+^ oxide throughout the XPS-related articles, only W_2_Cl_10_,^116,117^ we must estimate that the difference in the binding energy of our peaks and pure WO_3_ reference might justify, at least to some degree, the W^5+^ presence in our BCW samples.^118,119^ Nevertheless, the content of W ions surprisingly fits quite well to estimated Ce concentrations, roughly ∼24% for Ce^3+^ and ∼76% regarding Ce^4+^. This, in turn, somehow maintains the theory of 8+ B-site balance being kept throughout the sample. Quantitative discrepancies might be attributed to the technique’s sensitivity, fitting errors, or possible locally compensating, anisotropic defects which would not be detectable here, but still might upset Ce–W charge toward 7/9+ balance. Local Ce_7_O_12_ impurities might also be at play.

Following the BCW case, distorted ilmenite-like CCWO (top graph) has a much easier interpretation, considering that one of two transpiring sets of broad doublets has been seen previously identified during BCW analysis. The similarity and near-proximity (position differs by only 0.2–0.3 eV) of the first two peaks could be easily assigned again to W^5+^ as 4f_7/2_ near 34.6 eV and 4f_5/2_ at +2.1 eV. It stands for about ∼43% of the total registered tungsten. The other doublet, however, is different and spotted at much higher binding energy 4f_7/2_ = 36.4 eV and 4f_5/2_ = 38.1 eV. This concededly agrees with WO_3_ references hosting W^6+^ ions, suggesting that W^6+^ content would be remaining ∼57%. That concentration of specific tungsten species, again, does not deviate much from previous Ce 3d XPS results that predicted 59% of Ce^3+^and 41% of Ce^4+^. The theory here pursues, however, the 9+ balance at CCWO’s BB′-sites. To conclude, one more remark should also be made. Fwhm of all tungstate doublets is much bigger in our distorted ilmenite; therefore, one can anticipate different (likely worse) structural order inside the ilmenite than in ordered BCW double perovskite.^119−122^

**Figure 13:**
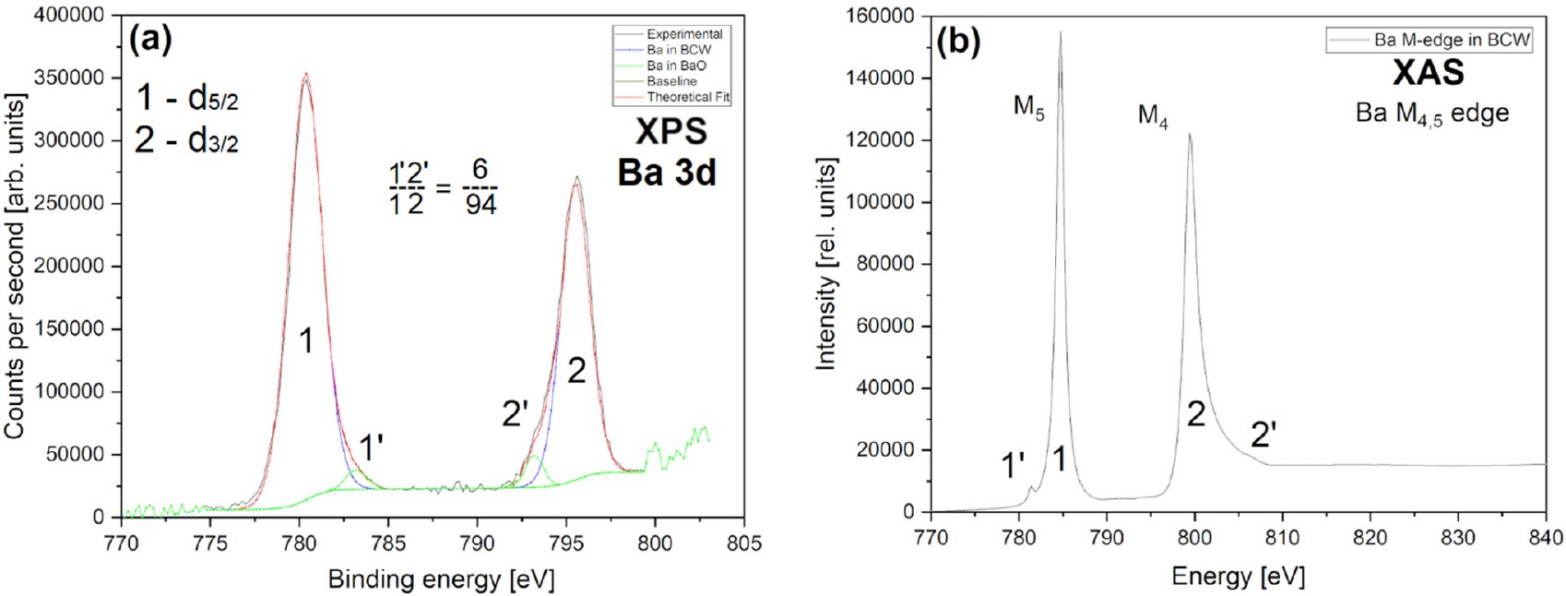
Barium 3d _5/2_ and d_3/2_ X-ray spectra presenting main and minor (impurity) Ba–O interactions within one of the purest batches of BCW. Graph (a) contains data for more surface-sensitive XPS, and (b) shows information about in-depth XAS M-edge measurement.

In Figure 13, XPS (a) barium 3d_5/2_, d_3/2_ spin–orbit doublet and XAS (b) barium M_4,5_-edge measurements for BCW look relatively similar. XPS 3d levels indicate that the main Ba^2+^ ionic state at BE (1) 780.4 eV and (2) 795.5 eV stands for 94% of total Ba atoms. Only ∼6% of them are stored in (1′) at 783.2 eV and (2′) 793.2 eV peaks; they are most certainly related to smaller (+1) ionic charges originating from local distortions and Ba–O impurities (matching the total content of 6% minor phases registered by XRD). In XAS, the M_4,5_ edge big peaks around (1) 785 and (2) 800 eV are analogically related to the same spin–orbit splitting of the main divalent ion. Barium oxide dodecahedra bond here to create our double-layered structure building main connections between Ce and W (···–O–Ce–O–Ba–O–W–O–···). Small features 1′ and 2′ (localized 3.4 eV apart, at the far beginning and end of each XAS band) are the same BaO/Ba–O–(Ce/W) related defects and impurities identified by XRD (Figure S5).^123−126^ Their partial separation from the hot, post-synthesis matrix (due to WO_2_ evaporation) could indicate unsuccessfully cerate formation via a BaO+CeO_2–*x*_ → BaCeO_3–*x*_ reaction.

**Figure 14:**
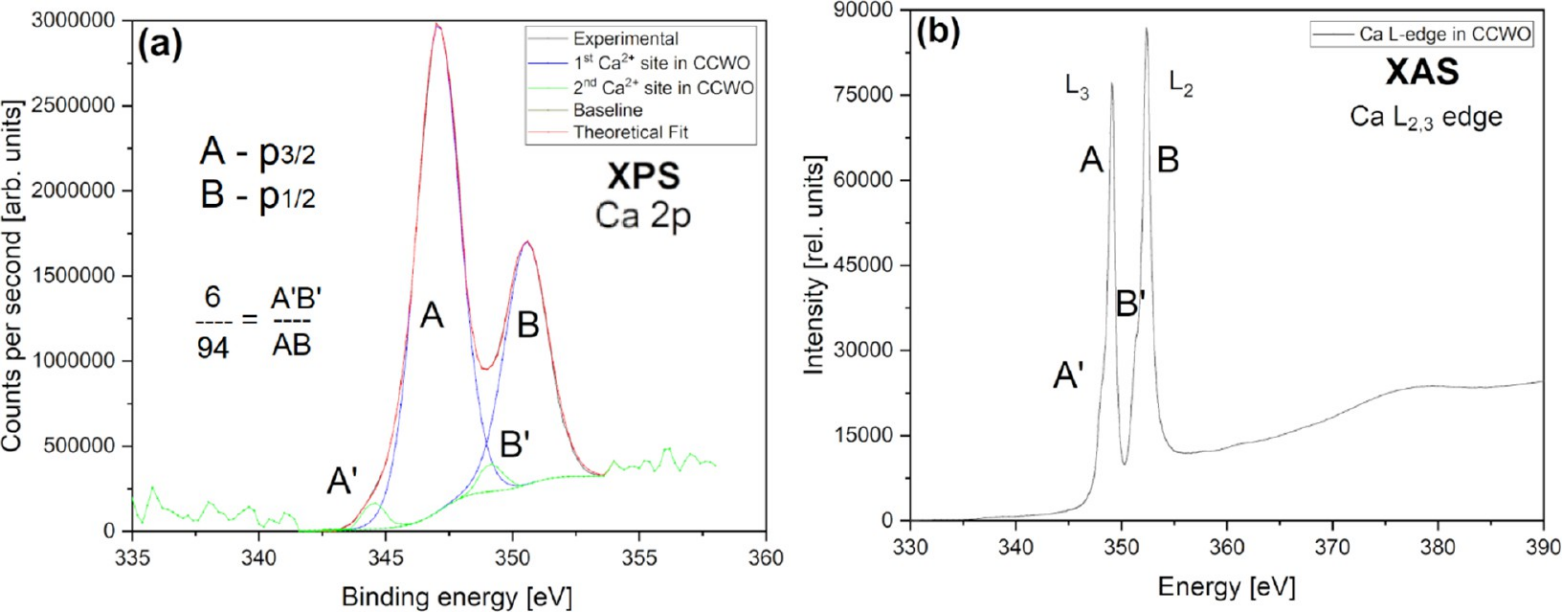
(a) In-depth XAS Ca L-edge measurements showing main symmetry-breaking interactions. (b) Surface-sensitive XPS spectra of Ca 2p_3/2_ and p_1/2_ within CCWO at room conditions and ultrahigh vacuum.

The calcium XPS 2p level with antibonding p_3/2_ and p_1/2_ states in Figure 14a has an analogical interpretation to convoluted XAS L_2,3_-edge spectra in Figure 14b, although those bands are a little bit more complicated to ascertain despite the high purity of the CCWO (which is ∼99% according to XRD; Figure 4a) since they are a bit different in matters of intensity and ratio. XPS Ca 2p spin–orbit doublets in Figure 14a have a weak resolution, but one can easily notice two bigger bands assigned to Ca^2+^ 2p_3/2_ (A) with BE 347.2 eV and lower BE 2p_1/2_ (B) at 350.7 eV. Together, they determine the main Ca site in CCWO consisting roughly 94% of total Ca content. In Figure 14b XAS, the concentration of the predominant phase is much lower, around 73% (A, 349.1; B, 352.4 eV), but the interpretation would be supported in the same way.^127,128^ Similarly detached XPS satellites (A′ + B′ features) consist of roughly 6% of the total batch; shoulders are vaguely separated by 2 eV. They are from some unique defective Ca(+1)–O bonding being probably also distinctly related to the aforementioned Ba–O in BCW, but here, they are separated by a wider 4.3 eV gap.^125,126,129,130^ Since those bands are much larger for XAS and are situated even closer to each other (being merely 1.5 eV apart) their interpretation is actually much more different. First, their contribution to the total signals stands around 27%. This is surely because of much deeper-situated symmetry-breakage typical for tilted ilmenite-like structures, not only from eventual impurities. As proven by XRD, in CCWO, one of three small alkaline Ca^2+^ atoms is trying to squeeze out from such a big unit cell partially swapping itself (sharing Wyckoff sites) with dominant Ce^3+^ ions. Bear in mind that we are actually missing that one calcium atom from the ideal double perovskite formula, Ca_3_Ce_2_W_2_O_12,_ which we were originally aiming for Ca_4_Ce_2_W_2_O_12_/2 = Ca_2_CeWO_6_; that one escaping atom is responsible for the ilmenite-tilting effect. Such a unique outcome would definitely impact Ca bonding — the changing hybridization would manifest itself somehow on the spectra. Similar cases were already reported by other scientists earlier.^3,9,131,132^ Considering that fact, partial Ca^2+^ (27% at best)/Ce^3+^ (57% of Ce^3+^ total content) substitution could really occur and the remaining ∼30 ± 2% of Ce^3+^ at standard CCWO BB′-sites would mix with their Ce^4+^ (∼41%) counterpart, thus creating many more charge-related defects with complementary bonded 5+/6+ tungstates. However, this problematic 9+ balance issue would require a separate, broad. nontrivial study which is not the main concern of that paper.

**Figure 15:**
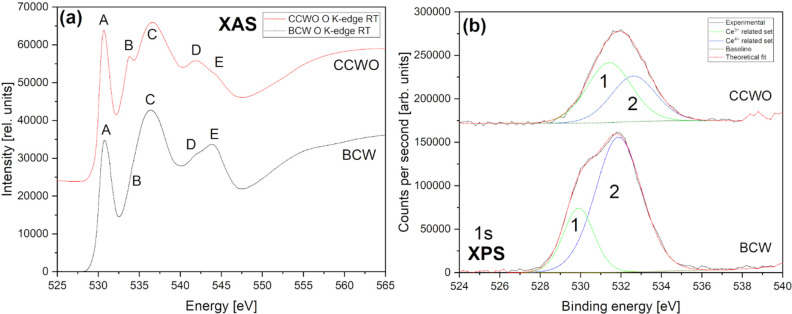
Oxygen K-edge X-ray spectra comparison between BCW (bottom) and CCWO (top) compounds collected at room temperature in an ultrahigh vacuum. Graph (a) shows complex XAS results with subtle but unique differences between each species, and (b) Much simpler XPS O 1s data deconvoluted into two straightforward sets of Ce^3/4+^-W^4/5/6+^ peaks.

Oxygen K-edge XAS spectra, in Figure 15a, reveal much more complex information about hybridization with rare earths or transition metals via sufficient DFT calculations—the latter is much easier said than done on structures such as DPs, as showcased by Groot et al.^133,134^ and Suntivich et al.^135^ Their methods were already pretty much convoluted for single perovskites having solely 1 d-block transition metal per simple, small unit cell. Here, results additionally enriched by rare earths, with possible minor phases (BCW), or tilted unit cell with partially substituted Wyckoff sites (CCWO) additionally complicate this theoretical approach, rendering them almost unintelligible to rationally fit. Due to the sheer complexity of our systems, we would like to focus only on a comparison between these two, unique XAS spectra to distinguish their main features by referencing respectable, supporting literature.

Let us first take into account that both BCW and CCWO exhibit more or less the same number of features on XAS but with a subtly different intensity ratio. Considering that fact, various O^2–^ ions interact with individual atoms in the same way but having different electronegativity due to spdf-block association, i.e., Ba/Ca–O, coexisting Ce^3/4+^–O, and W^5/4^ or W^5/6+^–O bonds. However, local environmental changes should also be observable and widely reflect various degrees of B-site covalent states substitution or shared Wyckoff site partial occupation. Unfortunately, often due to huge overlap in weakly responding structures it is risky to formulate some definitive answers. Namely, the difficulty arises from the fact that in order to see the difference, especially when the peaks are smeared and broad like here, one must perform meticulous deconvolution and relate discretely all O K-edge results to all previously collected data. That leaves us burdened with some inevitable errors.

In the case of BCW DP, the spectrum seems to show less detailed features related to overall ionic contribution. Maybe it is so because the structure is less complicated than that of CCWO ilmenite. All of the broad bands, seen after the abrupt onset, are actually posing as σ* bonds.^135^ Only the one at the beginning—(A) starting sharply around 530 eV—is considered antibonding π*.^136^ In our case, this signal has a weakly developed shoulder (being more pronounced in the case of CCWO) which might be also assigned to π* as an excitation of the O 1s core level toward hybridized, antibonding π* O 2p–chosen B-site d-molecular orbital. Since the coexistence of a second, nondominant B′-pair within each system is being considered, i.e., W^6/5+^ (in CCWO ∼6:4 ratio) or W^5/4+^ (in BCW ∼1:3), the presence of this second peak would be plausible. The aforementioned tungsten, coupled together with a respective Ce^3/4+^ 4f/5d pair (chosen in a fashion showcased earlier, maintaining 9+ charge balance characteristic for CCWO and 8+ for BCW) should form some pair of peaks in that region as visible energetic changes within their outer electron shells (orbitals) transpire.^135^ The aforementioned CCWO ilmenite additionally shows more distinctive, doubled features at higher energies—the (B) particular 533 eV set (virtually absent in BCW). It is typical for edge-sharing oxides, especially where local symmetry breakage within the nearest proximity to oxygen is present. Since calcium’s second nearest neighbor in the form of cerium (probably 3+) tries to partially substitute that alkali atom by squeezing into its dodecahedra, tilting it in the process, this phenomenon should be accounted for.^3,137^ It is worth mentioning that this signal is particularly pressure-sensitive and might correlate with the behavior of O 1s to O 2p–Ca4sp bands at higher energies when measured.^135,137^ That could theoretically be worth studying in the future in terms of possible *R*-3*c* → *C*2/*c* second-order phase transition above 9 GPa^68^ — visible on Raman spectra in Figures 8c and 9c.

BCW naturally has the aforementioned 533–534 eV features that are much weaker in that region due to their much smaller dislocation from the ideal, cubic *Fm*-3*m*, hence having higher symmetry (just 0.015° tilt toward *I*2*/m*) and lower concentration of the other, charge-differentiated (Δ = ±1 or 2) B-site oxidation pairs (aforementioned 1:3 ratio Ce^3+^/W^5+^ to Ce^4+^/W^4+^) which are also not shared nor partially substituted, contradicting CCWO, where cerium does openly try to switch with calcium. That is most likely because B-site ions in BCW possess a lower valence charge difference (in the minor Δ = ±1 and 0 in the major Ce/W phase). That should also somehow correlate with a much bigger electronegativity issue present in CCWO samples — the covalent character of Ce/W–O bonding in BCW is much more pronounced so, in consequence, energies of 4f–5d orbitals are closer to O 2p making classic hybridization much stronger.^135^ That would also explain the higher order in the latter sample.

In general, broad but distinctive σ* bands placed at (C) 534–538 eV tell us a bit more about interactions between alkaline atoms, oxygen, and d-block metal states such as 6s Ba/4s Ca–O–W 5d or 6s Ba/4s Ca–O–Ce 4f/5d. However, one must take into account that even if the current set of aforementioned peaks show any (slight) changes via displacement of O K-edge bands in comparison to the respective literature (due to the presence of tilt or not, due to partial substitution or lack of symmetry breakage), the differences viewed across A substitution sites at that range are still relatively small in comparison to much more pronounced B-site exchange. This fact should not surprise as the hybridization difference is mainly driven by the bonding angle of the central (BB′) atoms which are simultaneously controlling current bandwidths — those interactions are more spectacularly reflected when compared to XAS edge spectra of chosen RE or d-block ions.^135^ That fact alone, about the comparison of (shared or self-locked) O–B–O bonding interactions reflected in O K-edge spectra, sparked a discussion if there truly is a different, yet-unknown mechanism of charge transfer taking place in our samples, most likely related to some interstitial oxygen. This could somehow accommodate the reason why BCW internal charge transfer could be pushed thorough so far, but irreversibly. It does not share such features as CCWO, has fewer defects from spontaneous, chaotic, anisotropic pairing, and internal B-site ions are also less polarized; i.e., the PL-inactive Ce^4+^/W^4+^ pair has Δ*Z* = 0 in comparison to emitting Ce^3+^/W^5+^ Δ*Z* = 1.^138−142^ This subject will be divulged further in the upcoming optical paper concerning dopants and encountered luminescence issues.

Lastly, corner-sharing perovskite cells tend to have one of two reinforced oxygen bands visible between 540 (D) and 544 eV (E) due to self-achieved long-range periodicity or highly induced, short-range coordination associated with the formation of triply coordinated oxygen (like double triclusters of 2 × ABO_3_).^136,137^ This feature changes significantly from case to case, but as for CCWO having a very big, elongated rhombohedral unit cell in the shape of a tilted (a^–^a^–^a^–^) needle (*R-*3*c* of 342 atoms) the first peak would be definitely more pronounced than the latter in small but ordered BCW.

Figure 15b represents XPS data probing mainly unoccupied states of the O 2p conduction band for both investigated materials. They are quite simple in comparison to XAS. One can straightforwardly deconvolute the BCW spectrum into two single Gaussian-shaped components at BE 529.9 eV (1–25%) and 531.8 (2–75%). They can also be assigned to one of each dominating barium–oxygen-chosen Ce–W pair bridge. Explicitly, (1) to Ba_2_Ce^3+^W^5+^O_6_ and (2) to Ba_2_Ce^4+^W^4+^O_6_.^143,144^ Analogically, in the case of CCWO, at BE 531.4 eV (1–64%) and 532.6 eV (2–36%) to (1) predominant Ca_3_Ce^3+^_2_W^6+^_2_O_12_ and (2) less abundant Ca_3_Ce^4+^_2_W^5+^_2_O_12_.^145,146^ BaO impurities in BCW are probably hopelessly hidden or not even registerable in those circumstances due to high dilution (low concentration and weak sensitivity) within dualistic Ba–O-(Ce/W) matrix.

A brief summary of all collected XPS data (with signal positioning, characteristic assignments, and atomic content) is provided in Table 7 to calculate theoretic, empirical formulae of both investigated BCW and CCWO structures and confront them against the results provided by the XRD database. As can be seen, assumptions do not fall far behind initially established proportions: BCW = Ba_2_CeWO_6_ (being isostructural to Ba_2_BiYO_6_ on XRD) has an approximate formula of Ba_2.17_Ce_1.18_WO_6.81_. That results somehow support the theory of slow tungsten evaporation being in slight deficiency in comparison to all other recognized elements. Second, a slight surplus of oxygen matches the theory of additional, interstitial oxygen and vacancies pursued by the previous and upcoming chapter. Furthermore, if one hypothetically considers lingering 0.18 of Ce (1) and 0.17 Ba (2) we could also supposedly add them to a part of the rest remaining 0.81 oxygen — having something in the shape of already registered impurities (as a trial-and-error factor) such as Ba_0.17_O_0.17_ + Ce_0.18_O_0.36_ making something in the shape of Ba_0.17_Ce_0.18_O_0.53_ — that could make the apparent, excessive O content even smaller. CCWO = Ca_3_Ce_2_W_2_O_12_ being analogical to Ca_3_La_2_W_2_O_12_ has a stoichiometry of Ca_3.12_Ce_2_W_1.92_O_13.04_, meaning that the first two conclusions deducted previously regarding BCW (about W and O) also apply here.

**Table 7:** Summary of All Experimentally Investigated Core Levels on XPS, Their Content, And Theoretically Derived, Ideological Formulae for BCW (Normalized to W) and CCWO (Normalized to Ce) Based on Given Total Atomic Ratios

**Material**	**Core level**	**s–o split**	**Position (eV)**	**fwhm(eV)**	**Total atom (%)**	**Ratio**
BCW	W 4f	7/2	33.4, 34.4	1.65, 0.86	8.96	1
		5/2	35.4, 36.4	1.13, 0.88		
	O 1s		529.9, 531.8	1.78, 2.68	61.01	6.81
	Ba 3d	5/2	780.4, 783.2	2.21, 1.47	19.46	2.17
		3/2	793.2, 795.5	1.16, 1.88		
	Ce 3d	5/2	880.6, 883.1, 886.2, 889.6, 896.0	1.08, 2.20, 2.56, 3.64, 1.03	10.57	1.18
		3/2	898.3, 900.2, 903.0, 905.2, 917.3	1.91, 2.63, 2.31, 3.09, 2.51		
CCWO	W 4f	7/2	34.6, 36.4	1.27, 1.96	9.56	1.92
		5/2	36.5, 38.1	1.39, 1.76		
	Ca 2p	3/2	345.8, 347.2	1.23, 2.00	15.56	3.12
		1/2	349.2, 350.7	1.03, 1.94		
	O 1s		531.4, 532.6	1.59, 2.60	64.92	13.04
	Ce 3d	5/2	880.5, 883.4, 885.8, 889.0, 896.8	3.53, 3.66, 2.26, 2.85, 3.17	9.96	2
		3/2	899.8, 902.3, 904.4, 907.6, 915.4	3.22, 3.73, 3.02, 3.07, 2.84		

### 3.6 EPR vs Photoluminescence

**Figure 16:**
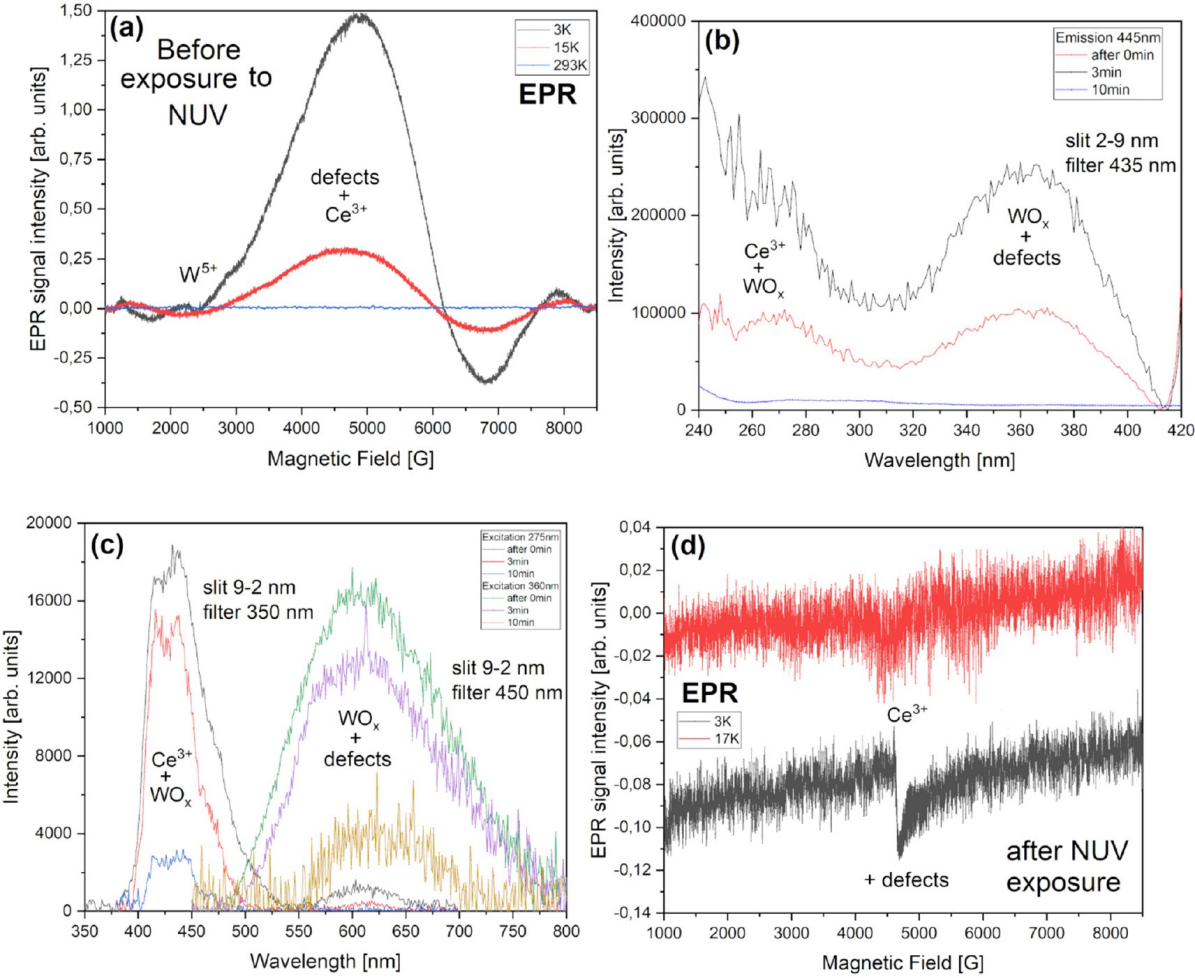
Results of low-temperature, magnetic and optical studies of charge transfer and photobleaching phenomena in BCW. (a) EPR spectra before exposure to NUV illumination, (b) PL excitation spectra taken on the pristine sample, during, and after exposure to NUV light. The duration of NUV illumination is given in the legend. (c) emission spectra during NUV exposure, and (d) EPR spectrum taken after last step of NUV illumination.

**Figure 17:**
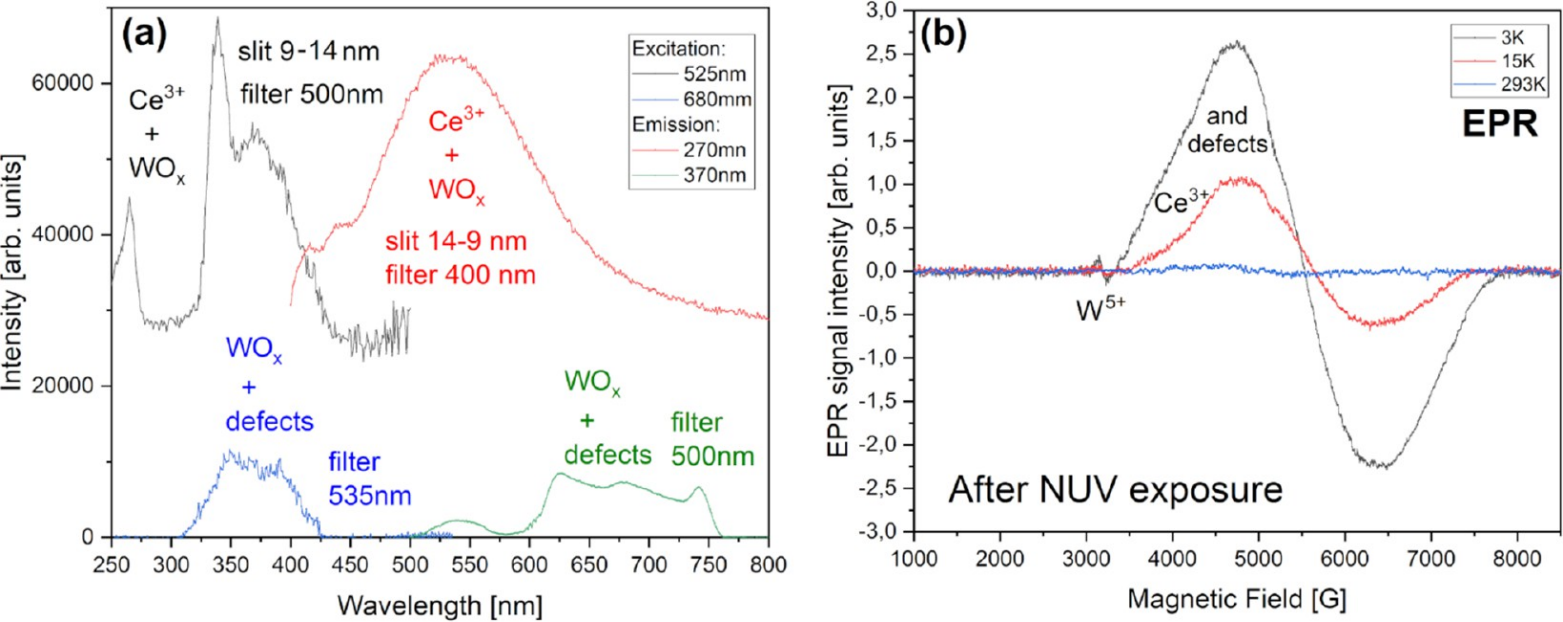
(a) Excitation and emission spectra of CCWO. (b) Low-temperature EPR spectra taken after NUV exposure.

EPR spectra shown in Figure 16 and Figure 17 provide complementary results for the registered ionic content determined by XPS and XAS. Among the magnetically active elements, we can also detect nonintentional ions present inside BCW and CCWO. We can definitely prove the existence of Ce^3+^ inside the predominant Ce^4+^/W^4+^ matrix of BCW. Namely, the intense, broad signal in Figure 16a visible below 20 K is a characteristic powder spectrum of half-spin rare-earth ions occupying several anisotropic, magnetically nonequivalent sites.^147,148^ However, apart from that unique cerium signal, we do not see any clear signal from coexisting W^5+^ions, which are naturally occurring, d-block charge-compensators, according to XPS data in Figure 12d. The signal should appear at about 3400 G owing to the *g*-factor = 1.989.^149,150^ It may be covered by the stronger Ce^3+^ signal since W^5+^ concentration is low. Since the material is diluted paramagnet with anisotropic (Ce–O, W–O) defects scattered randomly throughout the microcrystalline lattice^151^ and these ions are also PL-active, a parallel experiment was launched to assess their behavior. Progress was made by noticing that prolonged exposure of BCW to the NUV light expunges the purplish (blue + red) emission in a matter of minutes (with sunlight, several days). The best excitation wavelengths to observe such phenomena are ∼275 (Ce^3+^ + WO_*x*_) or 360 nm (WO_*x*_ + oxygen-related defect absorption bands) according to Figure 16b,c. The suggested physicochemical origins of those bands were discussed in a few sources.^9,152−155^ However, the deeper the excitation the faster our emission disappeared. Even after moving back to optimal wavelengths (from, i.e., 212 nm) the registered emission was hard to trace. Repeated EPR measurement in Figure 16d, carried out postfactum on the same illuminated BCW sample, shows the same effect. Where the intense, broad signal was only a very weak, narrow Ce^3+^ peak remained. It is important to pinpoint that while the aforementioned time-resolved PL gradually decayed in intensity, suggesting that already small Ce^3+^ content has been depleted, nothing visually changed in our sample. On XRD diffractograms, however, just after the experiment, small peaks from highly distorted CeO_2-x_ became a bit sharper and ordered in such a fashion that they resembled pure CeO_2_ (Figure S14a). Moreover, XPS showed only one set of predominant 3d Ce^4+^ and 4f W^4+^ peaks^101^ prompting, in comparison to previous results in Figure 12, that the recipient of transferred electrons from changing Ce^3+^ would most likely be W^5+^ as seen in Figure S14b,c.

Since the initially recorded PL irrecoverably disappeared and did not reemerge later on, even after several days, an irreversible photobleaching must have taken place within BCW. NUV and magnetically active Ce^3+^/W^5+^ pairs unrecoverably turn into inactive Ce^4+^/W^4+^ couples mitigating all energy conversion efforts. But, this could be useful regarding other technical (sensory) applications.^8,156^ Regarding CCWO, which contains mainly Ce^3+^ and W^6+^ ions, it seems that this material does not exhibit such behavior despite: showing the same, weak luminescence also influenced by oxygen vacancies (Figure 17a) and hosting similar, broad EPR Ce^3+^ signals characteristic for the predominant phase.^9,150−153^ Here, we can also see a visible weak W^5+^ signal persisting to 298 K (Figure 17b) with a *g*-factor around 1.973. CCWO also emits stable but weak light, unaffected by NUV illumination within a respective time frame. This suggests that the minor Ce^4+^/W^5+^ phase within it is stable and no charge transfer toward the main Ce^3+^/W^6+^ phase occurs and vice versa. This makes the BCW case even more anomalous. Since interstitial oxygen-bound defects can cause such effects^138−142^ we assume that Ba_2_PrWO_6_ could behave the same way.^157^

## 4 Conclusions

Two newly synthesized, undoped, oxide materials are reported: the ordered barium cerium-tungstate double perovskite Ba_2_CeWO_6_ (BCW) and ilmenite-like, disordered, calcium cerium tungstate Ca_3_Ce_2_W_2_O_12_ (CCWO). Both turned out to be weak luminophores. XRD investigations have shown that the BCW structure (∼95% pure) is better described by the (a^0^b^–^b^–^) space group (SG) *I*2*/m* having *a* = 6.0376(0) Å; *b* = 6.0369(0) Å; *c* = 8.5420(0)Å; with β angle being slightly tilted ∼90.015(0)° (0.04° at worst); rather than *R*-3 (*a*, *b* = 6.0381(8) Å; *c* = 14.7905(0) Å). The structure of CCWO (∼99% pure) is tentatively ascribed to the rhombohedral, centrosymmetric (a^–^a^–^a^–^) SG *R*-3*c* (*a*, *b* = 9.7258(3) Å; *c* = 55.2793(0) Å; α = 90°; β = 90°; γ = 120°) rather than *R*3*c* having *a*, *b* = 9.7258(2) Å; *c* = 55.2791(0) Å. However, this assignment is not final due to close similarities between the diffractograms (very close R factors for both SG) and incoherent behavior of various Raman scattering spectra at non ambient conditions. More HP XRD, and neutron diffraction measurements are required.

CCWO shows no clear change of polymorphism down to helium-level temperatures (∼5 K) based on Raman spectra, but it might undergo a second-order phase transition during the compression above 9 GPa to monoclinic *C*_*2*_*/c* SG. BCW, on the other hand, seems to be stable throughout high-pressure measurements up to 22 GPa (as *R*-3 should), but when cooled down below 200 K it could possibly undergo a second order phase transition between *R*-3 ⇄ *I*2*/m* SG. It is still uncertain if both phases could coexist with each other near or just below room temperature but the literature suggests that the latter phase would be more plausible at low temperatures due to monoaxial contraction. Unless it is a much sharper first-order phase transition between *I*2/*m* and *P*2_1_/*c* SG which can also occur at subzero temperatures. This would account for the larger number of peaks rapidly appearing at 150K and present itself as the main reason for choosing *I*2/*m* phase considering no access to more sophisticated, experimental techniques at that point.

Confronting the collected X-ray and spectral data with the additional statistical overview presented by Vasala and Karpinen^3^ in Figure S15 and Table S5, one could be more sure about XRD assignment of CCWO to *R*-3*c* and BCW to *I*2/*m* SG (close to cubic *Fm*-3*m* with angular tilt ranging from 0.04 to 0.015° depending on the batch).

As DSC, TG, and HT XRD investigations have shown, both compounds decompose quickly when heated in the air via two stages: above 573 K toward distorted ceria (CeO_2–*x*_ most likely being Ce_7_O_12_) due to Ce^3+^ oxidation; and above 773 K formation of tungstates (Ba/CaWO_*x*_) as W^4/5+^ starts to react. Further calcination forms mixed BaO or CaO oxides and tungsten oxides start to sublimate above 1373 K.

The PL photobleaching phenomenon was noticed solely in BCW during NUV PL measurements. Its origin is not fully understood yet, but connection to some kind of internal (probably interstitial) oxygen evolution and irreversible charge transfer within a relatively small unit cell has been noted using EPR and XPS.^116,138−142^

The latter technique, supported also by XAS, discovered while measuring the W 4f/O_2,3_-edge together with Ce 3d/M_4,5_. that both materials host mixed B-site charge states. Mainly, distorted CCWO is mixed in a 2:3 ratio hosting ∼59% mass of the predominant, photoactive Ce^3+^/W^6+^ pair, and the rest is Ce^4+^/W^5+^. That means that this particular ilmenite prefers a 9+ cationic balance. BCW is less diverse; it hosts only ∼24% mass of unstable Ce^3+^/W^5+^ photoactive couple with Ce^4+^/W^4+^ duo being the major part, therefore, maintaining a total of 8+ charge balance in a 1:3 ratio. While studying and comparing occupation sites, derived from XRD, together with XPS/XAS oxygen and alkali metal spectra (i.e., Ba 3d/M-edge; Ca 2p/L-edge, O 1s/K-edge) it can be assumed that no partial substitution of Ba/Ce ions is present in BCW in contrast to pronounced Ca/Ce exchange throughout various CCWO Wyckoff sites. This partial substitution with long crystallographic distances (inside the huge, complex unit cell of CCWO) was blamed for no observable photobleaching phenomena since the irregular current path would be obstructed by a lot of electron-capturing defects and abundant oxygen vacancies.

All these observations will be verified by upcoming, more detailed optical studies since this work only lays down fundamental, physicochemical research for much broader topics that we want to pursue later. Mainly, to implement those materials further in terms of downconverters or rather temperature/NUV sensors. This motivates the need for a more systematic and detailed investigation of selectively chosen RE dopants in the nearest future.

## Supporting Information Available

Macro photographs, XRD, XPS, Raman, and FTIR data obtained under various conditions (Figures S1–S15) along with five associated tables (Tables S1–S5) (PDF)
X-ray data for R-3_Ba_2_CeWO_6_ (CIF)
X-ray data for R3c_Ca_3_Ce_2_W_2_O_12_ (CIF)
X-ray data for R-3c_Ca_3_Ce_2_W_2_O_12_ (CIF)
X-ray data for I2m_Ba_2_CeWO_6_ (CIF)

## Supplementary Material

ao2c00669_si_001.pdf

ao2c00669_si_002.cif

ao2c00669_si_003.cif

ao2c00669_si_004.cif

ao2c00669_si_005.cif

## Abbreviations

EDTA: diethylenetriaminepentaacetic acid complex
DTPA: diethylenetriaminepentaacetic acid chelate
XRD: X-ray diffraction
XPS: X-ray photoelectron spectroscopy
XAS: X-ray absorption spectroscopy
EPR: electron paramagnetic resonance
FTIR: Fourier Transform Infrared
DSC: differential scanning calorimetry
TG: thermogravimetry
DPs: double perovskites
GS: Goldschmidt factor
mGS: modernized Goldschmidt factor
NUV: near ultraviolet
NIR: near infrared
CCD: charge coupled device
NA: aperture number
fwhm: full width at half maximum
Cp: heat capacity
SEM: scanning electron microscopy
HP: high pressure
DAC: diamond anvil cell
PTM: pressure transmitting medium
LT: low temperature
HT: high temperature
BCW: Ba_2_CeWO_6_
CCWO: Ca_3_Ce_2_W_2_O_12_
SG: space group
ICSD: inorganic crystal structure database
JCPDS: Joint Committee on Powder Diffraction Standards
RE: rare-earth (ions or atoms)
PL: photoluminescence
IR: infrared
DFT: density functional theory
BE: binding energy
