# Correction to “Synthesis Attempt and Structural Studies of Novel A_2_CeWO_6_ Double Perovskites (A^2+^ = Ba, Ca) in and outside of Ambient Conditions”

**DOI:** 10.1021/acsomega.2c08078

**Published:** 2023-01-09

**Authors:** Damian Wlodarczyk, Mikolaj Amilusik, Katarzyna M. Kosyl, Maciej Chrunik, Krystyna Lawniczak-Jablonska, Michal Strankowski, Marcin Zajac, Volodymyr Tsiumra, Aneta Grochot, Anna Reszka, Andrzej Suchocki, Tomasz Giela, Przemyslaw Iwanowski, Michal Bockowski, Hanka Przybylinska

The authors overlooked an unfortunate switch of (blue and green) colors between 2 neighboring peaks (v‴ and u_o_) in Figure 12a depicting the XPS Ce 3d shell on p 18396. The mistake was amended accordingly to fit the truth stated in the overall discussion (on p 18397) and provided calculations (p 18400). The error was purely visual in origin and only concerns the coloring within this one graph. All provided results, discussion, conclusions, and captions remain the same.

**Figure 12:**
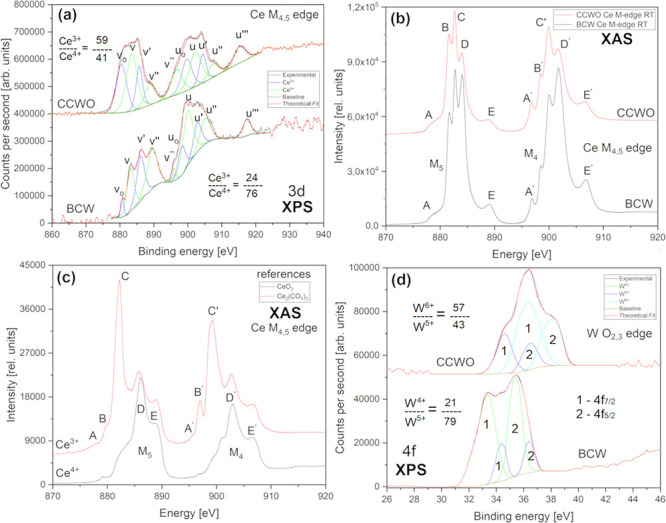
Crucial X-ray spectroscopy analysis collected at room temperature and ultra high vacuum for B-site cations, Ce, and W, present inside our powders and pellets showing the mixed, dualistic nature of both materials. The bottom curves mostly feature BCW and the top ones CCWO. Cerium 3d measurements for both surface-sensitive (a) XPS and more penetrating M-edge (b) XAS methods are presented as two top graphs for the respective samples. (c) has XAS spectra of pure 99+% references used for all syntheses. (d) presents the only available XPS 4f_7/2_ and 4f_5/2_ results regarding tungsten’s charge state doublets. XAS W edges were, unfortunately, out of the beamline energy range.

