# Supplementary material for: Synthesis Attempt and Structural Studies of Novel A2CeWO6 Double Perovskites (A2+ = Ba, Ca) in and outside of Ambient Conditions
Source: ACS Omega. 2022 May 23;7(22):18382–408. doi: 10.1021/acsomega.2c00669 (PMC9178617; doi:10.1021/acsomega.2c00669)
Supplement: Supplementary file 1 — ao2c00669_si_001.pdf [file ao2c00669_si_001.pdf]

Supplementary material

Synthesis attempt and structural studies of novel A_2CeWO_6 double perovskites (A^{2+}= Ba, Ca) in and outside of ambient conditions

**Damian Wlodarczyk[1], Mikolaj Amilusik[2], Katarzyna M. Kosyl[1], Maciej Chrunik[3], Krystyna Lawniczak-Jablonska[1], Michal Strankowski[4]. Marcin Zajac[5], Volodymyr Tsiumra[1], Aneta Grochot[1], Anna Reszka[1], Andrzej Suchocki[1], Tomasz Giela[5], Przemyslaw Iwanowski[1], Michal Bockowski[2], Hanka Przybylinska[1]*

[1]Institute of Physics, Polish Academy of Sciences, Ave. Lotnikow 32/46, PL-02668, Warsaw, Poland

[2]Institute of High Pressure, Polish Academy of Sciences, Sokolowska 29/37, PL-01142, Warsaw, Poland

[3]Military University of Technology, Gen. Sylwestra Kaliskiego 2, PL-00908, Warsaw, Poland

[4]Chemical Faculty, Gdansk University of Technology, G. Narutowicza 11/12, PL-80233 , Gdansk, Poland

[5]Solaris Synchrotron NSRC, Jagiellonian University, Czerwone Maki 98, PL-30392, Cracow, Poland

TABLE OF CONTENT:

2. Experimental S4

Figure S1 – Modified Vasala & Karppinen schemes S4

3. Results & Discussion S5

3.1 SEM imaging and morphology S5

Figure S2 – Macrophotograph of superheated CCWO S5

3.2 Powder XRD patterns in ambient conditions S6

Figure S3 – Case-specific XRD diffractograms SG fittings for Ba_2CeWO_6 S6

Table S1 – $Ca_3Ce_2W_2O_{12}$ structural details post Rietveld refinement S7

Table S2 – Rietveld R-factors for BCW and CCWO S7

Figure S4 – Detailed XRD diffractogram of main BCW minor phases S8

Figure S5 – XRD diffractogram fittings to Ce_7O_{12} S8

Figure S6 –XRD of all BCW recorded impurities S9

3.3 Raman & FTIR Spectroscopy S10

Table S3 – Group theory calculations S10

Table S4 – Ambient FTIR spectra characterization S11

Figure S7 – Raman spectra of impurity referrals S12

Figure S8 – Inert (N_2) temperature cascade Raman spectra S12

Figure S9 – Tilt systems phase transitions in double perovskites S13

3.4 High temperature studies – DSC, TG & XRD S14

Figure S10 – High-temperature XRD diffractograms of slow decomposition S14

Figure S11 – HighScore fittings of air-superheated, decomposed DPs S14

Figure S12 – High-temperature powder XRD structural dependencies of BCW S15

Figure S13 – High-temperature powder XRD structural dependencies of CCWO S16

3.6 EPR vs. photoluminescence S17

Figure S14 – EPR and XAS post-NUV evidence of photobleaching S17

4. Conclusions S18

Figure S15 – Statistics of recorded double perovskites, their GS and SG S18

Table S5 – Product placement of investigated compounds amongst DPs S19

2. Experimental

2.2 Synthesis Methodology

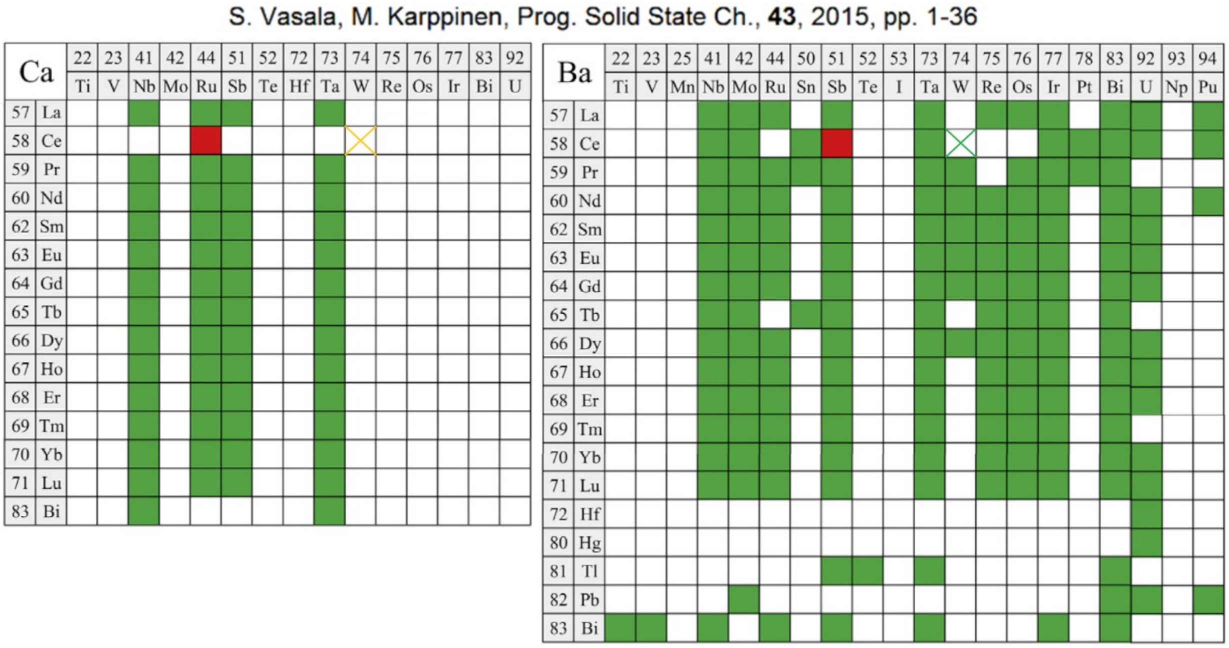

Figure S1 Modified, partially cropped statistical figures reported by Vasala and Karppinen[3] which gather information between the years 1950-2015 about created double perovskites in the region of interest - where our novel materials should also be placed. According to the authors: green boxes depict successfully synthesized DPs; red are failed attempts; White - not yet discovered materials; missing purple were those materials which were made at high-pressures; and yellow – ilmenites and other quasi-perovskite structures mostly in hexagonal or rhombohedral phase; Two new "X" placed artificially in the schemes with specific colors mark analogically our materials in the missing white slots. This graphic was adapted and reprinted in part with permission from Vasala S.; Karppinen M. $A_2B'B''O_6$ perovskites: A review, *Prog. Solid. State Ch.* **2015**, *43* (1-2), 1-36. Copyrighted and licensed by *Elsevier* Publisher via *Progress in Solid State Chemistry* journal since year 2015.

3. Results and Discussion

3.1 SEM imaging and morphology

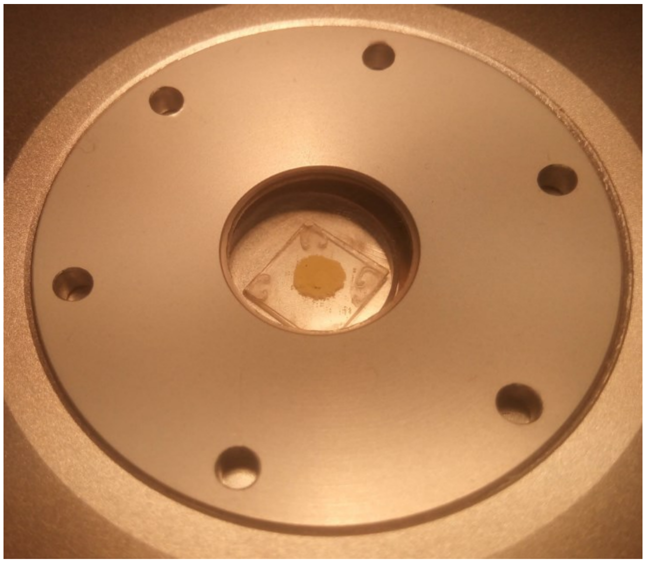

Figure S2 Macro photograph of superheated (to 873K) CCWO sample inside high-temperature LINKAM chamber. It has decomposed in air and was originally brown. It has turned into a yellowish-white mix containing mainly CeO_2 and $CaWO_4$.

3.2 Powder XRD patterns in ambient conditions

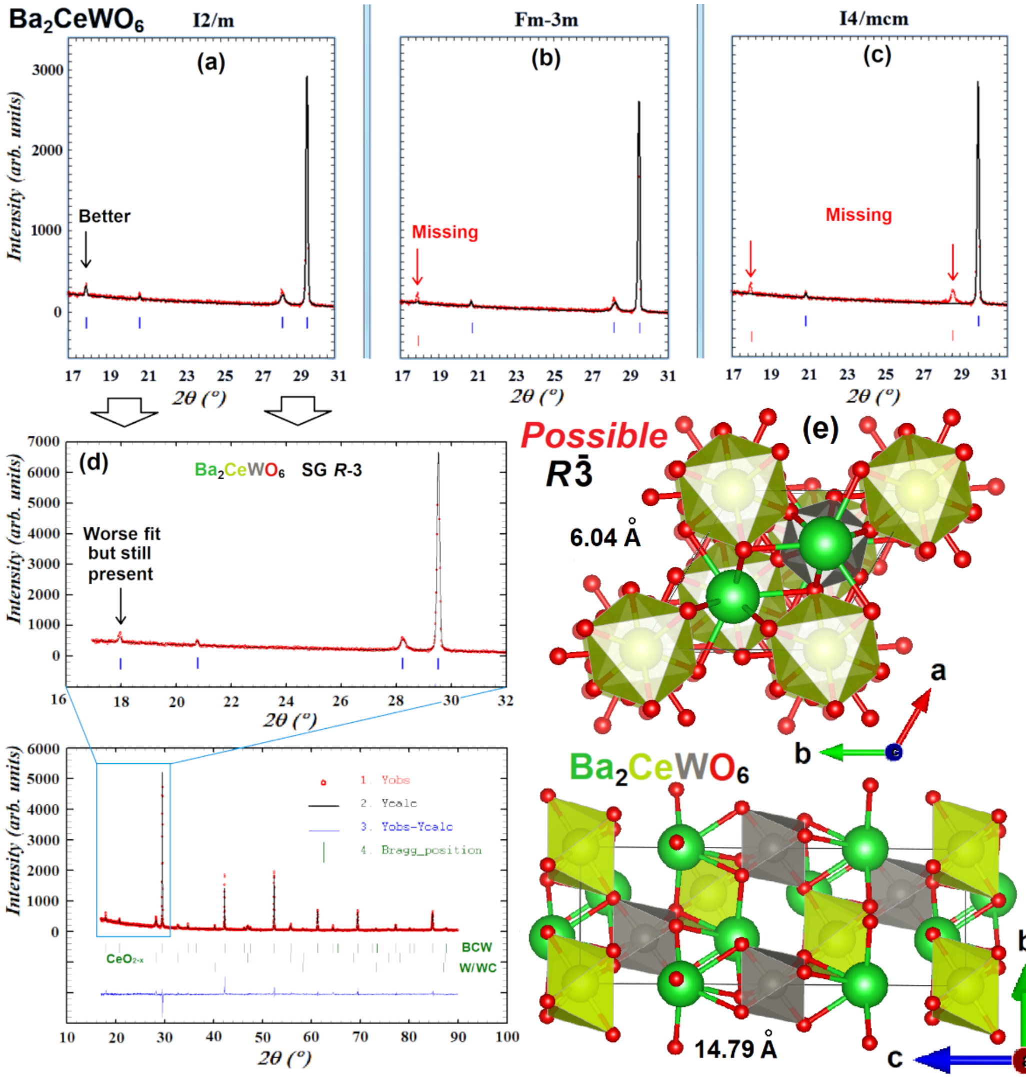

Figure S3 Case-specific powder XRD diffractograms showcasing a meticulous fitting process for the most possible SGs hosting BCW in a narrow 2θ region of interest. Red dots are the manifestation of a real, observed pattern; a straight black line is a calculated fit; blue and red vertical lines depict respectively present and missing Bragg positions (green lines) in comparison to chosen *I*2/*m* SG. As one can see, pattern (a) for *I2/m* contains all 4 peaks, while cubic *Fm*-3*m* at (b) and *I*4*/mcm* in (c) came short of 1 or 2 positions respectively. Figure (d) also matches all of them but *hkl* at 18° (2θ) lacks in matters of intensity – this pattern is associated with alternative *R-3* unit cell which is presented in mixed polyhedral/ball-stick convention in Figure (e).

Table S1 Structural details of new $Ca_3Ce_2W_2O_{12}$ structure, based on the Rietveld refinement. Chosen, preferable space group: *R*-3*c*, a = 9.7258(3) Å, c = 55.2793(0) Å,V = 4528.41(8) Å^3. Note that U_{iso} has been set to be the same for all O and W atoms, respectively.

Site	*x*	*y*	*z*	U_{iso} [Å^2]	Occ. (<1)
Ca1	0.0093(2)	0.35914(19)	0.18694(2)	0.0033(4)	0.678(2)
Ce1	0.0093(2)	0.35914(19)	0.18694(2)	0.0033(4)	0.322(2)
Ca2	0.37275(16)	0.04466(14)	0.04948(2)	0.0017(3)	0.5541(19)
Ce2	0.37275(16)	0.04466(14)	0.04948(2)	0.0017(3)	0.4459(19)
Ca3	0	0	0.10415(3)	0.0007(6)	0.303(6)
Ce3	0	0	0.10415(3)	0.0007(6)	0.697(6)
Ca4	0	0	0.25	0.043(2)	
W1	0.33174(11)	0	0.25	−0.00009(14)	
W2	0	0	0.162812(18)	−0.00009(14)	
W3	0	0	0	−0.00009(14)	
O1	−0.0035(14)	0.1863(10)	0.22340(15)	0.0025(9)	
O2	0.0036(13)	0.1713(11)	0.02094(14)	0.0025(9)	
O3	0.1355(10)	0.1911(11)	0.17969(16)	0.0025(9)	
O4	0.1863(10)	0.0745(11)	0.14022(15)	0.0025(9)	
O5	0.1927(10)	0.3107(12)	0.10760(14)	0.0025(9)	
O6	0.2460(8)	0.1049(9)	0.0836(2)	0.0025(9)	

Table S2 Conventional Rietveld reliability factors for alternative BCW and CCWO SG and their diffractograms hosting all present Bragg contribution patterns corrected with the background.

Formula	SG	Z	V [Å^3]	d_{cal} [g/cm^3]	R_B	R_P	R_{WP}	R_{EXP}	N_σ GoF	χ2	Fract [%]
Ba_2CeWO_6	*R*-3	3	467.009	7.410	13.6	23.7	20.9	4.41	1295.277	23.0	94.73
CeO_{2-x}	*Fm*-3*m*	4	164.207	6.962	42.3						2.41
BaO	*Fm*-3*m*	4	168.007	6.062	37.0						2.55
W(/WC)	*Im*-3*m*	2	31.698	19.263	93.8						0.31
$Ca_3Ce_2W_2O_{12}$	*R*3*c*	18	4528.403	6.339	4.95	8.89	10.4	2.70	900.651	14.8	99.61
W(/WC)	*Im*-3*m*	2	31.678	19.257	70.8						0.39

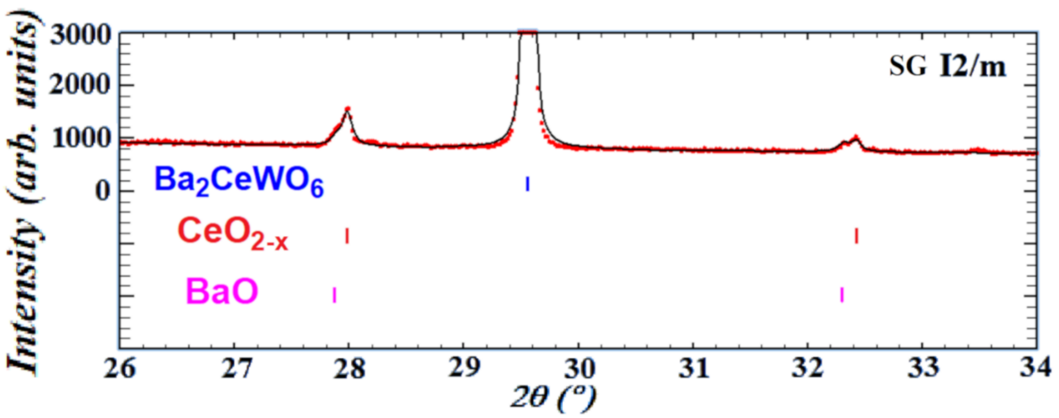

Figure S4 Powder XRD diffractogram showcasing a typical *I*2/*m* fit for one of the worse BCW batches. As can be seen, quite a few minor phases could form within the nearest proximity of nearby Bragg positions, such as here, highly-distorted CeO_{2-x} and BaO. Red dots are the manifestation of a real, observed pattern; a straight black line is a calculated fit; and the blue, red, and pink vertical lines relate to specific compounds and their Bragg positions – BCW, CeO_{2-x}, and BaO respectively. Those impurities form due to the evaporation of 1 important reactant - WO_2.

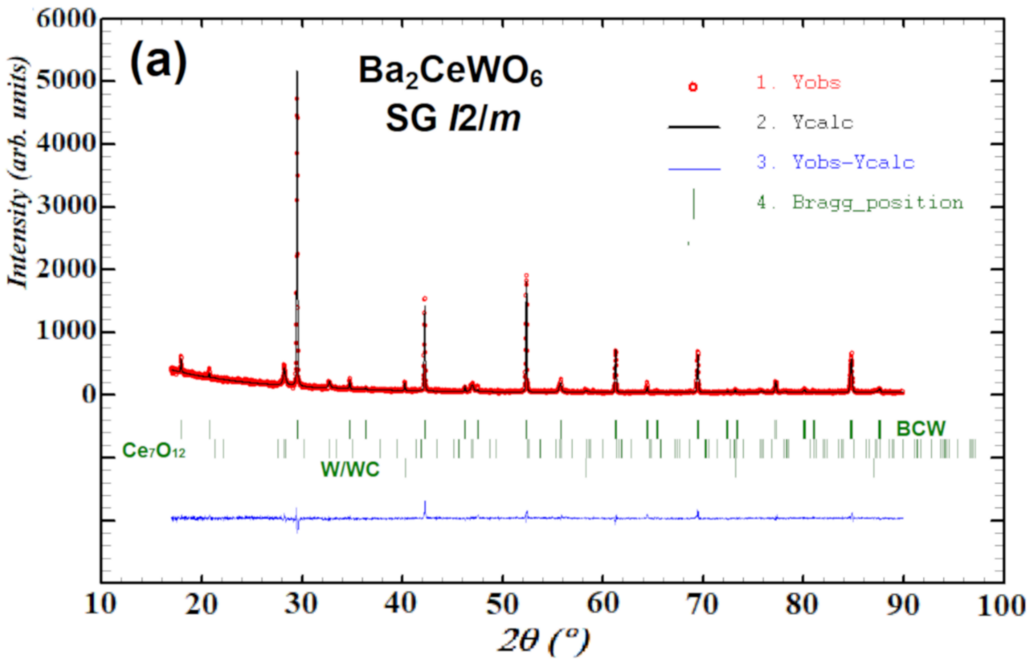

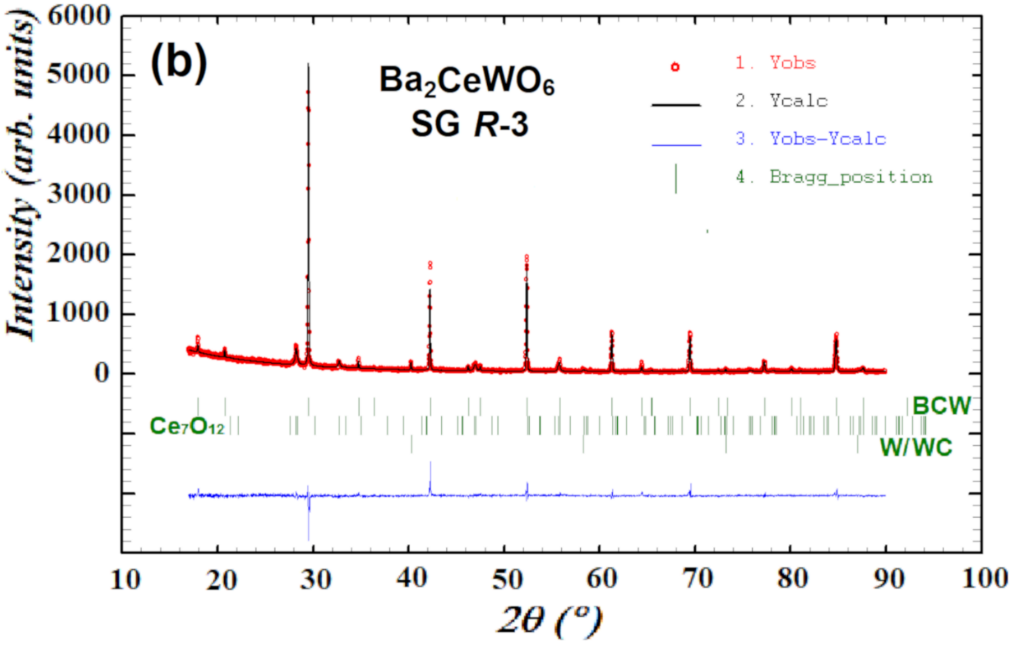

Figure S5 Powder XRD diffractogram fit for one of the better BCW batches showing that in a few particular cases a minor phase such as highly-distorted CeO_{2-x} could form an almost perfect Ce_7O_{12} pattern. It is probably a targeted form of this undesired impurity concerning almost identical: (a) *I*2/*m*, and (b) *R*-3 SG patterns. Red dots are the manifestation of a real, observed pattern; a straight black line is a calculated fit; and the green vertical lines relate to specific compounds and their Bragg positions.

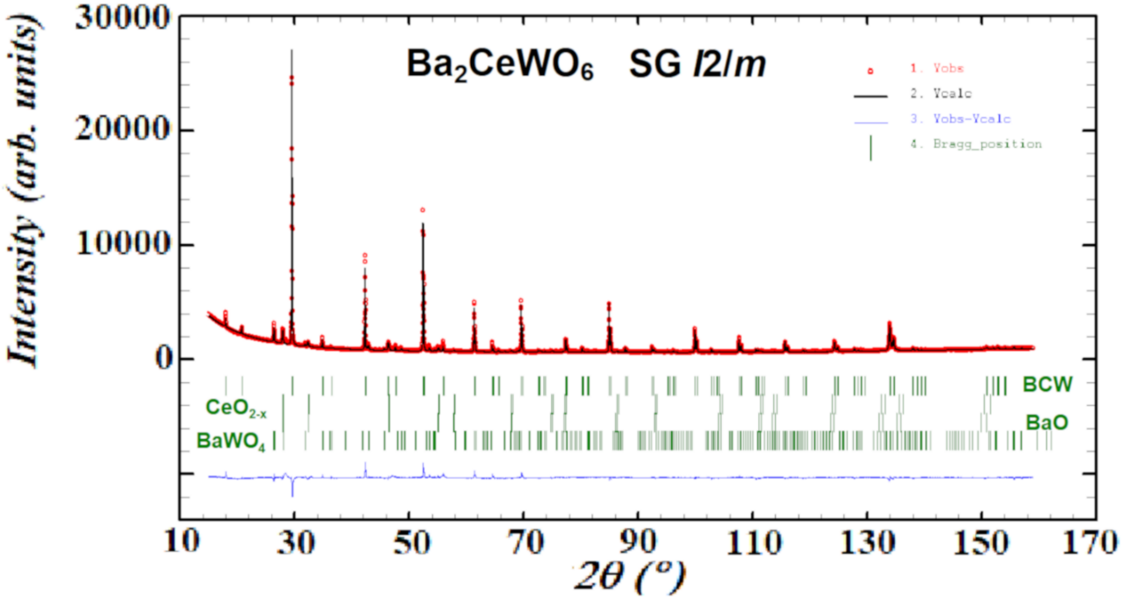

Figure S6 Powder XRD diffractogram fit for one of the worst BCW batches showing that quite a few minor phases could form: not only highly-distorted CeO_{2-x} and BaO present due to WO_2 evaporation, but also quite current $BaWO_4$ created when an excessive amount of air is not removed from the furnace environment. Red dots are the manifestation of a real, observed pattern; a straight black line is a calculated fit; and green vertical lines relate to specific compounds and their Bragg positions – BCW, CeO_{2-x}, BaO, and $BaWO_4$.

3.3 Raman & FTIR Spectroscopy

Table S3 Factor group analysis for relevant space groups assigned to both CCWO and BCW.

Material (SG)	Ion	Wyckoffs Positions	Point Symmetry	Red. Representation
**BCW** ***I2/m*** ***(C_{2h})*** ***Assumed***	A^{2+} (Ba)	4i	C_s	$2A_{1g}+A_u+B_g+2B_u$
	$B^{3+/4+}$ (Ce)	2a	C_{2h}	A_u+2B_u
	$B'^{4+/5+}$ (W)	2d	C_{2h}	A_u+2B_u
	O_1^{2-}	4i	C_s	$2A_{1g}+A_u+B_g+2B_u$
	O_2^{2-}	8j	C_1	$3A_{1g}+3A_u+3B_g+3B_u$
$\Gamma_{TOTAL} = 7A_{1g}+7A_u+5B_g+11B_u$; $\Gamma_{ACUSTIC} = A_u+2B_u$; $\Gamma_{IR} = 6A_u+9B_u$; $\Gamma_{Raman} = 7A_{1g}+5B_g$				
**BCW** ***R-3*** ***(C_{3i})*** ***Possible***	A^{2+} (Ba)	2c	C_3	$A_g+A_u+{}^1E_u+{}^2E_u+{}^1E_g+{}^2E_g$
	$B^{3+/4+}$ (Ce)	1a	C_{3i}	$A_u+{}^1E_u+{}^2E_u$
	$B'^{4+/5+}$ (W)	1b	C_{3i}	$A_u+{}^1E_u+{}^2E_u$
	O^{2-}	6f	C_1	$3A_g+3A_u+3{}^1E_u+3{}^2E_u+3{}^1E_g+3{}^2E_g$
$\Gamma_{TOTAL} = 4A_g+6A_u+6{}^1E_u+6{}^2E_u+4{}^1E_g+4{}^2E_g$; $\Gamma_{ACUSTIC} = A_u+{}^1E_u+{}^2E_u$; $\Gamma_{IR} = 5A_u+5{}^1E_u+5{}^2E_u$; $\Gamma_{Raman} = 4A_g+4{}^1E_g+4{}^2E_g$				
**CCWO** ***R-3c*** ***($D3_d$)*** ***Assumed***	A^{2+} (Ca)	2a, 4c, 12f	C_1, C_3, D_3	$4A_{1g}+5A_{2u}+9E_u+9E_g$
	$B^{3/4+}$ (Ce)	4c, 12f	C_1, C_3	$4A_{1g}+4A_{2u}+8E_u+8E_g$
	$B'^{6/5+}$ (W)	6e, 4c, 2b	C_2, C_3, C_{3i},	$2A_{1g}+4A_{2u}+7E_u+5E_g$
	O^{2-}	12f	C_1	$3A_{1g}+3A_{2u}+6E_u+6E_g$
$\Gamma_{TOTAL} = 13A_{1g}+28E_g+16A_{2u}+30E_u$; $\Gamma_{ACUSTIC} = A_{2u}+E_u$; $\Gamma_{IR} = 15A_{2u}+29E_u$; $\Gamma_{Raman} = 13A_{1g}+28E_g$				
**CCWO** ***R3c*** ***(C_{3v})***	$B^{6/5+}$ (W)	2a, 6b	C_1, C_3	$4A_1+8E$
	$B'^{3/4+}$ (Ce)	2a, 6b	C_1, C_3	$4A_1+8E$
	A^{2+} (Ca)	2a, 6b	C_1, C_3	$4A_1+8E$

Possible	O^{2-}	6b	C_1	$3A_1+5E$
$\Gamma_{TOTAL} = 15A_1+29E$; $\Gamma_{ACUSTIC} = A_1+E$; $\Gamma_{IR} = 7A_1+14E$; $\Gamma_{Raman} = 7A_1+14E$				

Unfortunately, regarding FTIR, only a few, convoluted peaks are visible. What should be a rich spectrum of $6A_u+9B_u$ IR active modes in BCW; or 7A+14E in CCWO (according to assumed SGs from group theory calculations in Table S3) is most likely cut out from characteristic fingerprint pattern due to the machine's limited capabilities. [74-79] FTIR apparatus could not easily reach the range below 400 cm^{-1} without additional assets like a pumped N_2-pure environment. This is problematic, especially considering the same breadth and convolution of nearby peaks from defects and various BB'-sites. Therefore because not much can be said and done with great detail about the whole spectral region we have decided to primarily focus on Raman spectra analysis and present just this crude Table S4 for future reference. If the SG for BCW is *R*-3 IR modes A_u and B_u should be substituted with A_u and E_u, respectively.

Table S4 Conjoined experimental data and literature phonon assignment collected in ambient conditions using confocal FTIR spectroscopy for both BCW (*I*2/*m*) and CCWO (*R*-3*c*) compounds emphasizing simultaneously observable differences and similarities.[74-79]

Wavenumber [cm⁻¹] ω_0	***Compound & characteristics***	***Assignment***
476 ; 459	BCW & CCWO m, sh	In- and out-of-plane bending bands region for both WO_6, and partially substituted CeO_6 clusters of B_u/E_u origin down to 100 cm^{-1}.
554 ; 560	BCW & CCWO s, sh	ν_{sym} stretch of both ions in WO_6, and partially substituted CeO_6 clusters of A_u/A_{2u} origin.
632 ; 636	BCW & CCWO m, sh	
722	CCWO mw, sh	ν_{asym} stretch of both ions in WO_6, and partially substituted CeO_6 clusters of A_u/A_{2u} origin.
855	CCWO w, sh	

w-weak; m-medium; s-strong; sh-sharp; br-broad; ν_{sym} – symmetric stretching; ν_{asym} – asymmetric stretching;

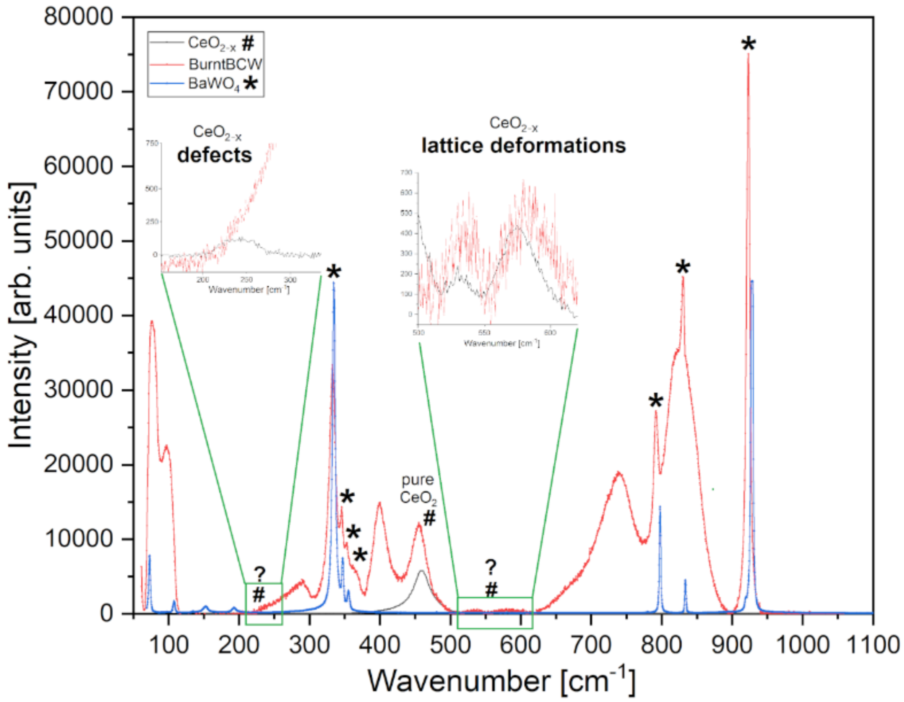

Figure S7 Raman spectra showcasing popular impurity referrals such as CeO_{2-x} (black) and $BaWO_4$ (blue lines) in contrast to one of the better BCW batches (red) burnt in ambient conditions during exposition to the excessive power of the green laser light. Transpiring phases alongside their characteristic modes are pinpointed via hashtags (CeO_{2-x}), and asterisks ($BaWO_4$) respectively to guide the eye.

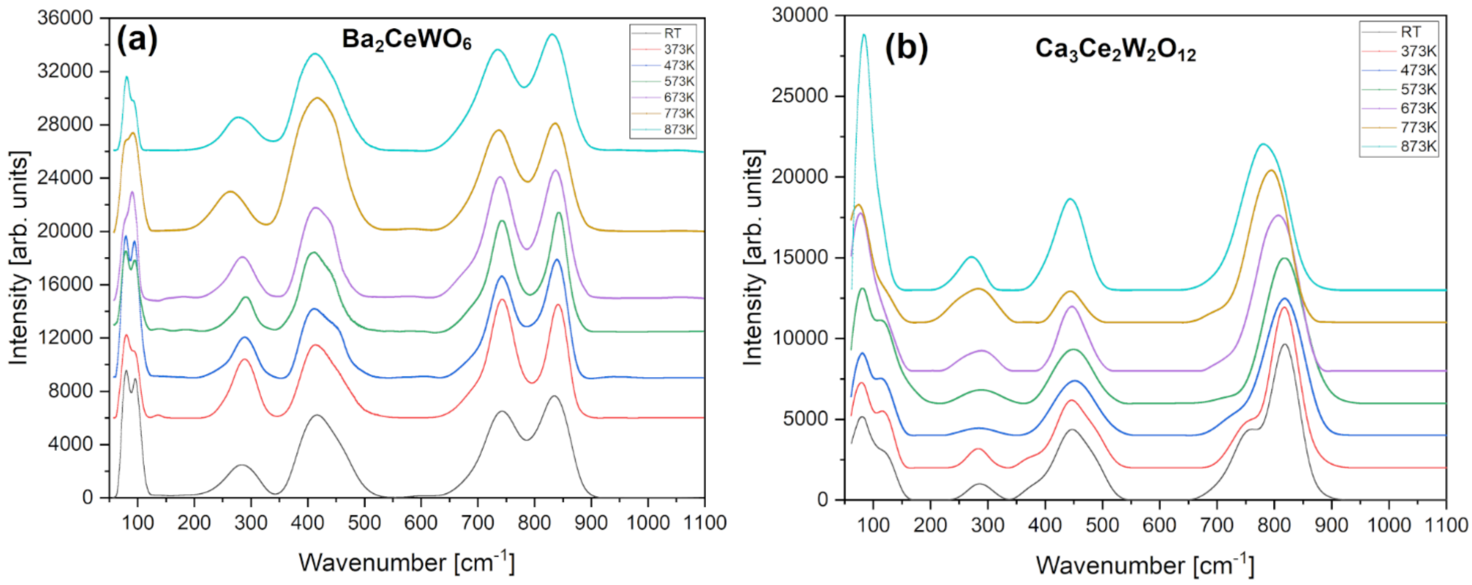

Figure S8 Raman spectra for both (a) BCW, and (b) CCWO collected in the function of heating from room temperature up to 873K in protective, inert N_2 atmosphere showing little to no change - only slight blue-shift with amorphization were noticed.

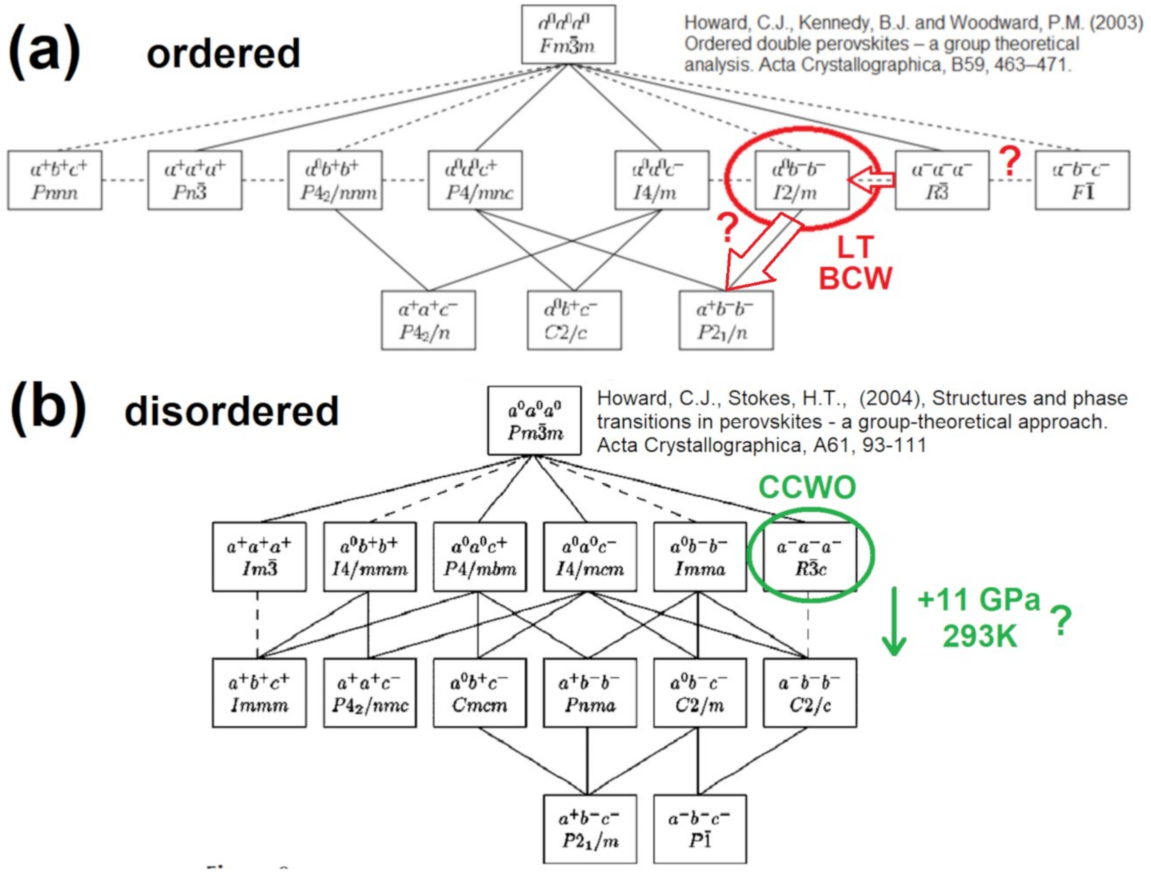

Figure S9 Group–subgroup relations associated with particular tilt systems phase transitions in $A_2BB'X_6$ (a) ordered (like BCW), and (b) disordered perovskites-like materials (similar to CCWO) evaluated by Howard et. al. between 1998-2005 using DFT calculations.[90,91] Continuous black lines are first-, and dashed second-order transformations. Possible sample placement has also been established artificially amongst possible SG. Both graphics were adapted and reprinted in part with permissions from Howard C. J.; Kennedy B. J.; Woodward P. M. Ordered double perovskites – a group-theoretical analysis, *Acta Crystallogr.* **2003**, *B59*, 463-471, and Howard C. J.; Stokes H. T. Structures and phase transitions in perovskites – a group-theoretical approach, *Acta Crystallogr.*

2004, *A61*, 93-111. Copyrighted and licensed by *IUCr Journals* Publisher via *Acta Crystallographica B* journal after 2003 & 2004 respectively.

3.4 High-temperature studies – DSC, TG & XRD

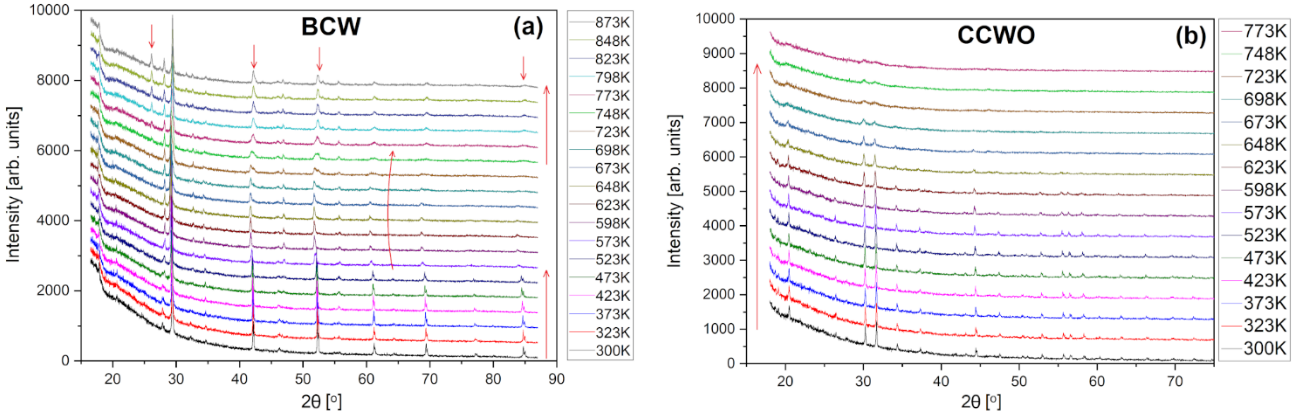

Figure S10 High-temperature powder XRD diffractograms presented in the function of slow heating for (a) BCW, and (b) CCWO respectively. Changes in them prove gradual degradation and continuous amorphization of both materials – red arrows guide the eye in terms of a few, key-specific features.

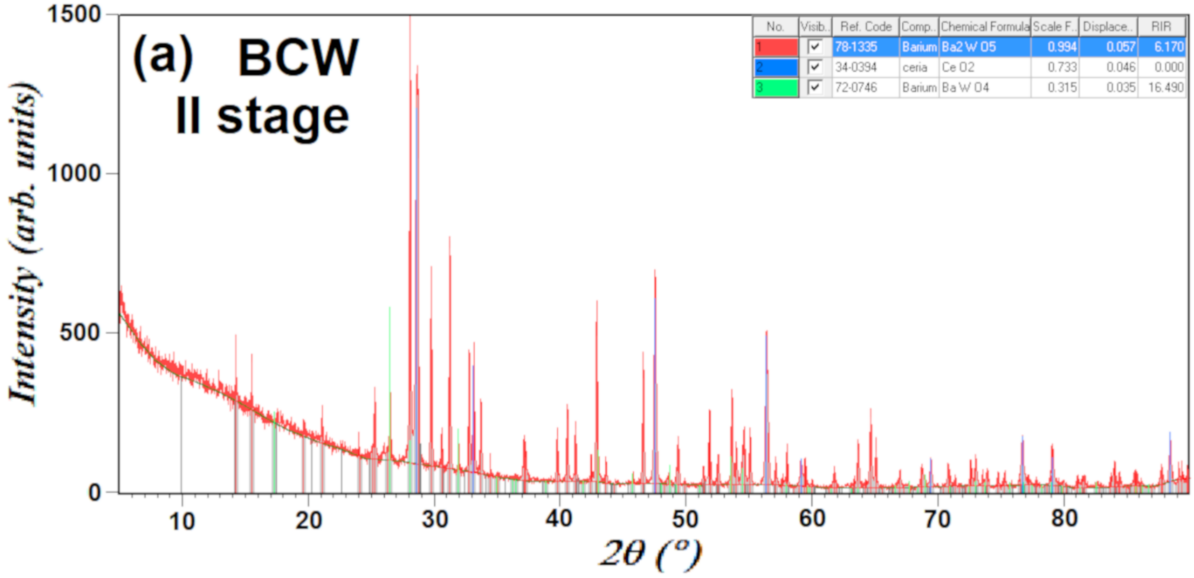

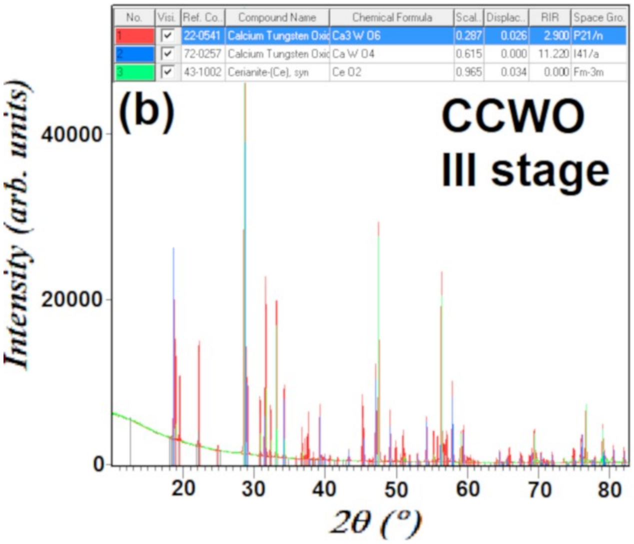

Figure S11 Powder XRD spectra of both (a) BCW, and (b) CCWO heated to II and III stage of synthesis temperatures but in the air, without any protective, inert atmosphere. They showcase prominent decomposition to CeO_{2-x} and respectable tungstates via quick HighScore database fitting.

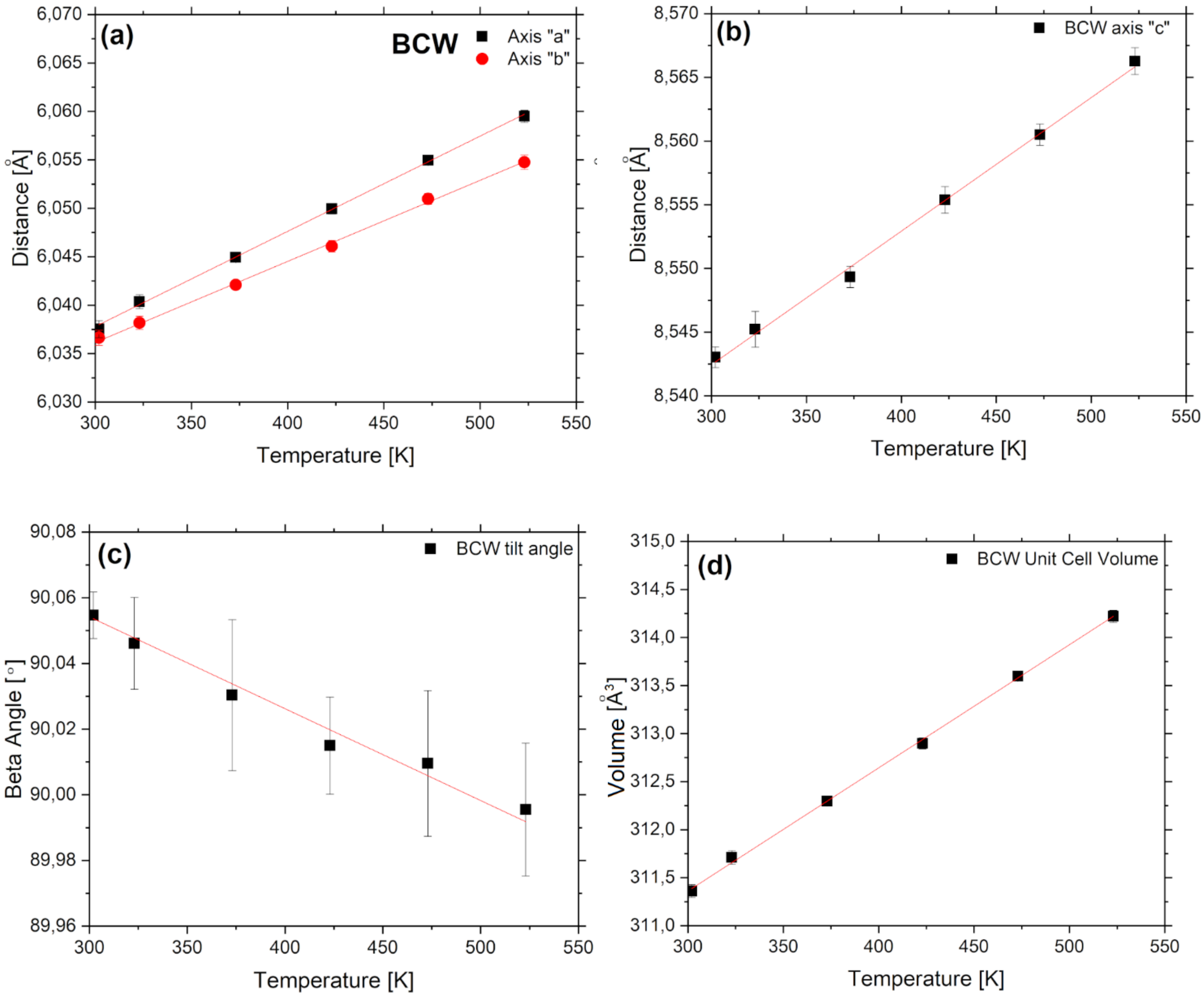

Figure S12 Various structural dependencies were determined through high-temperature powder XRD measurements in a novel, Ba_2CeWO_6 unit cell. The crystallographic structure was investigated while heating in the air before any possible signs of decomposition was noted. Figure (a) covers expansion along similar "*a*" and "*b*" axes while (b) presents changes in a bit longer "*c*" dimension. Graph (c) showcases a particular case of angular tilting being diminished towards perfect, right angle while on (d) an expected, progressive, volumetric expansion can be seen. Red lines are linear fits after which mean values presented in Table 6 were determined.

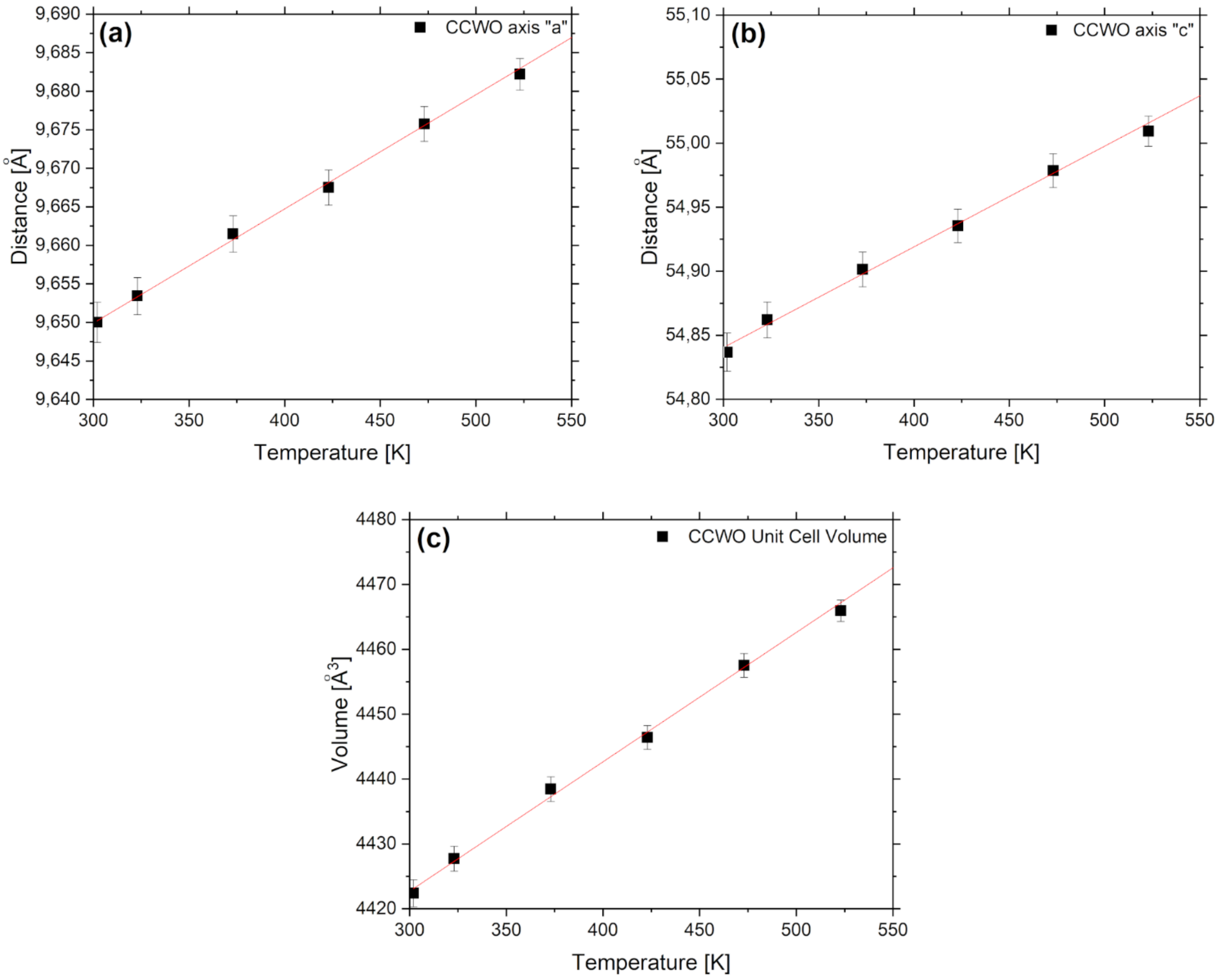

Figure S13 Various structural dependencies determined through high-temperature powder XRD measurements in the novel $Ca_3Ce_2W_2O_{12}$ unit cell. The crystallographic structure was investigated while heating in the air before any possible signs of decomposition was noted. Figure (a) covers expansion only along "*a*" axis since "*b*" is considered obsolete in *R*-3*c* SG; (b) presents changes in a bit longer "*c*" dimension. Graph (c) showcases an expected progressive volumetric expansion during the experiment. Red lines are linear fits after which mean values presented in Table 6 were determined.

3.6 EPR vs. photoluminescence

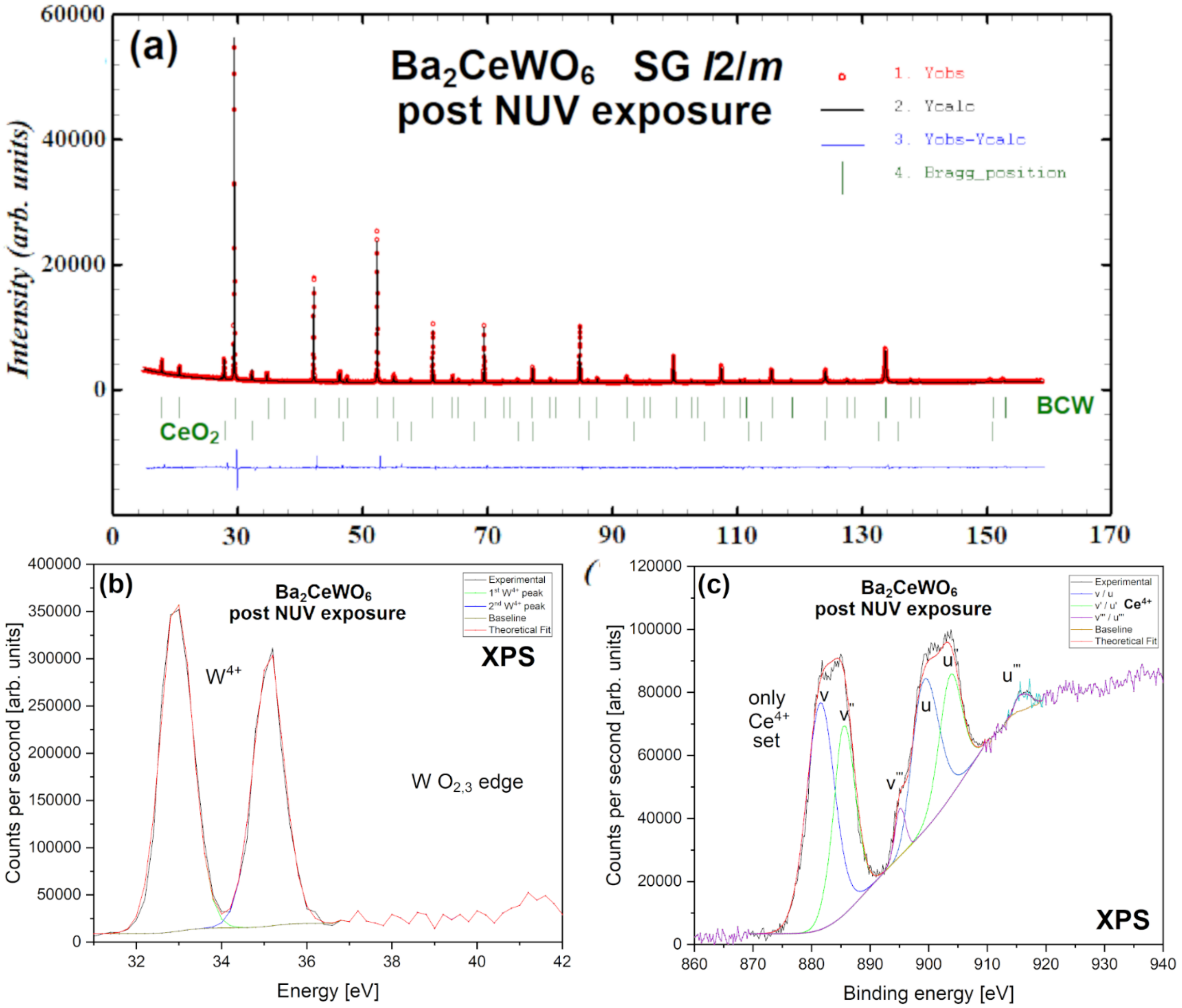

Figure S14 A brief overview of main BCW structure post NUV exposure, after so-called photobleaching occurred. As one can see on (a) powder XRD diffractogram the main phase remained vaguely the same alongside with classic, not-distorted CeO_2 impurity. The only observable XPS (b) tungsten $O_{2,3}$-edge doublet and (c) Ce 3d multiplet signify the sole presence of 4+ pairing within the compound after this brief exposure.

4. Conclusions

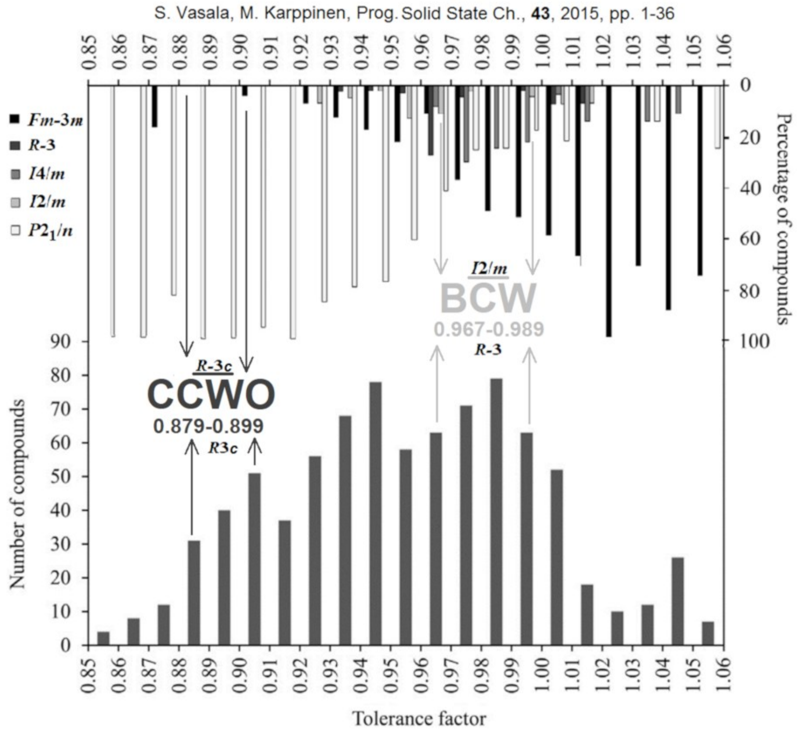

Figure S15 A comprehensive summary of DP studies in recent, 65 years (1950-2015) performed by Vasala and Karppinen[3] showing: the number of $A_2BB'O_6$ compounds reported with different values of GS tolerance factor, and the percentages of these materials reported with a specific space group. A crude placement of both newly synthesized products within those statistics has been performed. Two edge-limit positions have been implemented depending on $Ce^{3/4+}$: $W^{4/5/6+}$ ion content. Such wide range-explanation is necessary since there is a mixture of both depending on the source – BCW $Ce^{3/4+}$ vs $W^{5/4+}$; and CCWO $Ce^{3/4+}$ vs $W^{6/5+}$. This graphic was adapted and

reprinted in part with permission from Vasala S.; Karppinen M. $A_2B'B''O_6$ perovskites: A review, *Prog. Solid. State Ch.* **2015**, *43* (1-2), 1-36. Copyrighted and licensed by *Elsevier Publisher* via *Progress in Solid State Chemistry* journal since year 2015.

Table S5 Most common space groups and tilt systems, for both ordered and disordered double-perovskite-like structures, were reported by Vasala and Karppinen[3] till 2015 with artificial addition of our newly synthesized materials. The Red color depicts our final assignments. Green, with asterisks, is a possible option also mentioned by the original authors. This table was adapted and reprinted in part with permission from Vasala S.; Karppinen M. $A_2B'B''O_6$ perovskites: A review, *Prog. Solid. State Ch.* **2015**, *43* (1-2), 1-36. Copyrighted and licensed by *Elsevier Publisher* via *Progress in Solid State Chemistry* journal since year 2015.

**Glazer tilt**	**Space Group**		**Number of compounds**	
$a^0a^0a^0$	*Pm*-3*m*		11	
$a^-a^-a^-$	*R*-3*c*	+CCWO	5+1	
$a^0a^0c^-$	I4/mcm		4	Disordered Perovskites (Ilmenites)
$a^0b^-b^-$	*Imma*		1	
$a^-b^+a^-$	*Pnma*		45	
$a^+b^-c^-$	*P*2$_1$/*m*		1	
$a^0a^0a^0$	*Fm*-3*m*		146	
$a^-a^-a^-$	*R*-3*	+1	19*	
$a^0a^0c^-$	*I*4/*m*		44	
$a^0a^0c^+$	*P*4/*mnc*		0	Ordered Perovskites
$a^0b^-b^-$	*I*2/*m*		19+1	
$a^+b^-b^-$	*P*2$_1$/*n*		310	
$a^-b^-c^-$	*I*-1		2	
